# Pediatric primary central nervous system germ cell tumors of different prognosis groups show characteristic miRNome traits and chromosome copy number variations

**DOI:** 10.1186/1471-2164-11-132

**Published:** 2010-02-24

**Authors:** Hsei-Wei Wang, Yu-Hsuan Wu, Jui-Yu Hsieh, Muh-Lii Liang, Meng-En Chao, Da-Jung Liu, Ming-Ta Hsu, Tai-Tong Wong

**Affiliations:** 1 School of Life Sciences, Institute of Microbiology and Immunology, National Yang-Ming University, Taipei, Taiwan; 2 School of Medicine, Institute of Clinical Medicine, National Yang-Ming University, Taipei, Taiwan; 3 School of Life Sciences, Institute of Biochemistry and Molecular Biology, National Yang-Ming University, Taipei, Taiwan; 4 VGH Yang-Ming Genome Research Center, National Yang-Ming University, Taipei, Taiwan; 5 Department of Education and Research, Taipei City Hospital, Taipei, Taiwan; 6 Division of Pediatric Neurosurgery, Neurological Institute, Taipei Veterans General Hospital, Taipei, Taiwan

## Abstract

**Background:**

Intracranial pediatric germ cell tumors (GCTs) are rare and heterogeneous neoplasms and vary in histological differentiation, prognosis and clinical behavior. Germinoma and mature teratoma are GCTs that have a good prognosis, while other types of GCTs, termed nongerminomatous malignant germ cell tumors (NGMGCTs), are tumors with an intermediate or poor prognosis. The second group of tumors requires more extensive drug and irradiation treatment regimens. The mechanisms underlying the differences in incidence and prognosis of the various GCT subgroups are unclear.

**Results:**

We identified a distinct mRNA profile correlating with GCT histological differentiation and prognosis, and also present in this study the first miRNA profile of pediatric primary intracranial GCTs. Most of the differentially expressed miRNAs were downregulated in germinomas, but miR-142-5p and miR-146a were upregulated. Genes responsible for self-renewal (such as POU5F1 (OCT4), NANOG and KLF4) and the immune response were abundant in germinomas, while genes associated with neuron differentiation, Wnt/β-catenin pathway, invasiveness and epithelial-mesenchymal transition (including SNAI2 (SLUG) and TWIST2) were abundant in NGMGCTs. Clear transcriptome segregation based on patient survival was observed, with malignant NGMGCTs being closest to embryonic stem cells. Chromosome copy number variations (CNVs) at cytobands 4q13.3-4q28.3 and 9p11.2-9q13 correlated with GCT malignancy and clinical risk. Six genes (BANK1, CXCL9, CXCL11, DDIT4L, ELOVL6 and HERC5) within 4q13.3-4q28.3 were more abundant in germinomas.

**Conclusions:**

Our results integrate molecular profiles with clinical observations and provide insights into the underlying mechanisms causing GCT malignancy. The genes, pathways and microRNAs identified have the potential to be novel therapeutic targets.

## Background

The reported incidence of primary germ cell tumors (GCTs) of central nervous system (CNS) in children is significantly higher in Taiwan, Japan and Korea compared to Western countries. The comparative incidences are 15.3% in Japan, 14.0% in Taiwan, 11.2% in Korea, 2.3% in USA, and 2.5% in German in various reported series [1-5]. There is still no explanation for this extreme geographic and ethnic difference between the three Asian series and the two Western series (*p *< 0.0001) [5]. Genomic differences need to be considered and evaluated.

Primary CNS GCT consists of several subtypes with different degrees of histological differentiation and malignancy. According to histological differentiation, related tumor markers, and secreted protein markers, these tumors can be classified into germinomas and nongerminomatous GCTs (NGGCTs), the latter including embryonal carcinoma (EC), yolk sac tumors (YST), choriocarcinoma (CC), teratoma (mature teratoma, immature teratoma, or immature teratoma with malignant differentiation) and mixed GCTs [6]. For NGGCTs, except for benign mature teratoma, all of the other tumors present with diverse malignancies and therapeutic sensitivities when compared to germinomas and are grouped together as nongerminomatous malignant GCTs (NGMGCTs). NGMGCTs require more extensive drug and irradiation treatment regimens, have a higher recurrence rate and a lower survival rate [7,8]. Clinically, >50% of pediatric CNS GCTs are germinomas, while the majority of remaining tumors are NGMGCTs [5,9]. Histologically, germinoma is the most undifferentiated GCT and is composed of undifferentiated large cells that resemble primordial germinal elements. Among the NGGCTs, the histological picture differs depending on the diagnosis. EC contains undifferentiated stem cells resembling the embryonic inner cell mass (ICM). YST and CC correspond to the extra-embryonic differentiation along mesoblast and trophoblast lines, respectively. This contrasts with teratomas, which consist of differentiated derivatives that include all three germ layers with or without incompletely differentiated tissue elements, like neuroepithelium, which resembles fetal tissue. CNS GCTs often present with more than one histological component and are then classified as mixed GCTs [7,10,11].

GCTs are presumed to arise from mutated primordial germ cells (PGCs) of genital ridge origin or dysfunction totipotent embryonic cells [12]. Investigation of the different genetic compositions in ECs and ES cells may provide clues about the reduced dependency on external cues for self-maintenance that exist among GCTs, thereby benefiting tumorigenesis research on ECs as well as applications for human ES cells (see also a review article by Werbowetski-Ogilvie *et al*. [13]). Global gene expression studies in human embryonic stem cells and human pluripotent germ cell tumors have shown that the gene expression patterns of human ES cell lines are similar to those of the human embryonal carcinoma cell samples but are more distantly related to those of seminoma samples [12,14]. Genes that are expressed at significantly greater levels in human ES and embryonal carcinoma cell lines than in control samples were pinpointed and are possible candidates for involvement in the maintenance of a pluripotent undifferentiated phenotype [12]. Wnt and Notch pathway genes are overexpressed in the pluripotent human embryonal carcinoma cell line NTERA2 and in embryonic stem cells [15]. These include members of the frizzled gene family (FZDI, FZD3, FZD4, FZD5, FZD6), which encodes receptors for the Wnt proteins, the Frizzled Related Protein family (SFRPI, SFRP2, FRZB, SFRP4), which encode soluble Wnt antagonists and also ligands and receptors of the Notch pathway (Dlkl, Jaggedl; Notchl, Notch2, Notch3) [15].

The histological differences between the various different GCTs are mirrored by their gene expression profiles [16,17]. Genomic studies have been conducted on GCTs, most notably on Caucasian adult gonadal ones [12,16]. However, only limited gene profiling studies have focused on primary pediatric CNS GCTs, and, to our knowledge, no transcriptome profiling work on Asian cases has been reported. A very recently paper studied global mRNA expression patterns in pediatric malignant GCTs arising from the testis, the ovary, the sacrococcygeal region and the brain, and then compared these with adult testicular tumors. These results showed that there is no segregation of GCTs with the same histology at different sites or at different ages, within the pediatric range. However, clear segregation of pediatric and adult tumors, most conspicuously among the YSTs, was observed [17]. The pediatric seminomas are significantly enriched for genes associated with a self-renewing pluripotent phenotype, whereas the pediatric YSTs are significantly enriched for genes associated with differentiation and proliferation [17]. These results suggest that the observed clinical differences between pediatric CNS GCTs from different ethnic backgrounds or prognosis groups may also be detected using genomic analysis.

MicroRNAs (miRNAs) are small RNAs of 18-24 nucleotides in length that are involved in the regulation of gene expression and hence a variety of biological processes through post-transcriptional RNA interference-based mechanisms. Matured miRNAs interact and inhibit target mRNAs and result in translational repression or mRNA cleavage [18-20]. In medulloblastoma (MB), an aggressive brain malignancy with a predominant incidence in childhood, a high throughput miRNA profiling analysis found that only a few miRNAs displayed upregulated expression, while most of them, such as miR-9 and miR-125a, were downregulated in the tumor samples, suggesting a tumor growth-inhibitory function [21]. Moreover, the same group identified miRNAs downregulated in human MBs with high Hedgehog (Hh) signaling, which is one of the pathogenesis mechanisms of MB [22]. Differential miRNAs, such as miR-184, have been identified and found to correlate with prognosis, differentiation, and apoptosis in pediatric neuroblastoma [23]. A high-throughput miRNome analysis of adult gonadal GCTs has been published, and in each GCT subtype the miRNA patterns are quite different [24]. For GCTs in children, only limited miRNA data has been reported.

Genomic copy number variation (CNV) in GCTs of adulthood has been extensively investigated. Gain of 12p in up to 80% of cases of adult testicular GCTs [25,26]. In contrast, comparatively little genomic CNV investigation has been conducted on childhood GCTs. Using metaphase comparative genomic hybridization (CGH), a wide range of CNVs has been described in pediatric GCTs, including gains on 1q, 2p, 3, 7, 8, 13, 14, 20q, 21, and X, as well as losses on 1p36, 4q, 6q, 11, 13 and 18; but none are seen consistently [27-29]. This may due to either the heterogeneity of the GCTs, or the different algorithms that were applied to identify the CNV regions. In 2007, Palmer *et al*. used 34 GCTs (22 yolk sac tumors (YSTs), 11 germinomatous tumors and one metastatic embryonal carcinoma), which had occurred in children from birth to age 16, for CNV analysis. Most of their cases were from the testis, the ovary and the sacrococcygeal region and only two germinomas and one YST brain BCT were included [30]. Gain of 12p was found to be present in 53% of primary MGCTs of children aged 5-16 and was also observed in four of fourteen YSTs affecting children less than 5 years old. The YSTs showed an increased frequency of 1p loss (*p *= 0.003), 3p gain (*p *= 0.02), 4q loss (*p *= 0.07) and 6q loss (*p *= 0.004) compared to the germinomatous tumors [30].

In this study, we applied genomic approaches to explore the molecular messages governing the ethnic and prognosis differences of CNS GCTs. Both mRNA and miRNome expression patterns were studied in pediatric primary CNS GCTs. To provide novel insights into GCT pathogenesis, the transcriptomes of all GCT cases were further compared to those of ES cell lines from both Caucasian and Taiwanese genetic backgrounds [12,23]. Copy number variations (CNVs) in different GCT subtypes were also measured to evaluate their possible influence on gene expression traits. Finally, the transcriptomes of our patients were organized into functional modules in order to identify the dominant biological processes and key genes in the germinomas and NGMGCTs; this sought to help explain the clinical observations associated with these tumors.

## Results

### Clinical aspects of primary pediatric CNS GCTs examined

In our series of 176 cases of primary pediatric CNS GCTs, 58.5% were germinoma and 41.5% were nongerminomatous GCTs (NGGCTs). Among the germinomas, 62.1% had a histological diagnosis, while the remaining 37.9% of cases had a presumptive diagnosis. For NGMGCTs, 90.3% had a histological diagnosis, with the remaining cases having a presumptive diagnosis. Each presumptive diagnosis of the GCTs was made according to the tumor's clinical features, neuroimaging results, serum tumor marker level (alpha fetal protein [AFP], beta human chorionic gonadotropin level [beta-hCG]) and response to radiotherapy and/or chemotherapy. Subtypes of NGGCTs included mature teratomas (5.1%), various NGMGCTs including immature teratomas, mixed GCTs, pure YSTs, and tumors diagnosed by tumor markers (35.2%), and unclassified GCTs (1.7%) (Additional file 1-A) The 5-year, 10-year and 15-year overall survival rates for the germinomas and NGMGCTs were 82.2%, 74.5% and 74.5% for the germinomas and 66.1%, 45.4% and 30.3% for the NGMGCTs.

**Figure 1:**
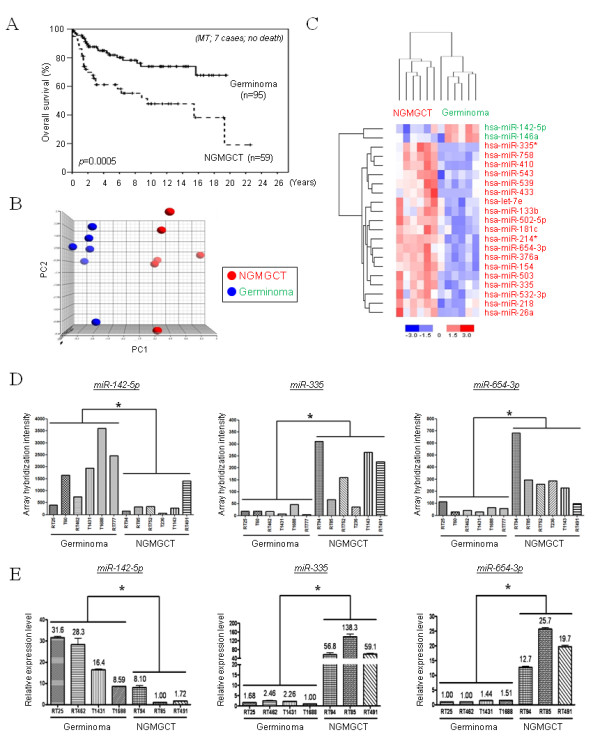
**MiRNome analysis of childhood CNS GCTs**. (**A**) Overall survival rates of GCTs of different histological subtypes. In total, 161 patients were followed up for up to 20 years. These were then subjected to Kaplan-Meier survival analysis. Numbers in parentheses are case numbers of each tumor subtype. Vertical lines indicate the censored survival observations. (**B**) Principal component analysis (PCA) using the filtered miRNAs (*p *< 0.05 and fold change ≧2). Each spot represents a single array. (**C**) A heat map shows the miRNAs enriched in the different prognostic groups. MiRNAs in red showed increased expression, while those in blue showed decreased. (**D**-**E**) Validation of miRNA array results by real-time PCR. The mean expression levels of the target miRNAs are compared to that of the U6 small nuclear RNA control. Results are expressed as the mean ± standard deviation (SD) (**E**). The miRNAs' array hybridization signals are also shown (**D**).

Kaplan-Meier estimator analysis and log-rank test revealed that the germinoma patients had a better overall survival than the NGMGCT patients (*p *= 0.0005; Figure 1A). Accordingly, therapeutic classification of the GCTs represents prognostic factor-based classification and management. However, the therapeutic classification of CNS GCTs is quite different between the CNS GCTs and extra-CNS GCTs, because of rareness of systemic metastasis of the CNS GCTs [9]. According to the clinical and therapeutic classification of CNS GCTs [9], in our series of CNS GCTs in children, 113 cases (63.6%), including 103 germinomas, 9 mature teratomas, and 1 mixed germinoma and mature teratoma, were categorized as members of the good prognostic group (GPG), 40 cases, including 12 immature teratomas and 19 mixed GCTs, were categorized as members of the intermediate prognostic group (IPG), and 14 cases, including 10 pure yolk sac tumors and 4 mixed GCTs dominated by yolk sac tumors, were categorized as members of the poor prognostic group (PGG) [9]. For the 21 cases that underwent genomic studies (Additional file 1-B), cases 1-12 could be categorized as members of the GPG and these included 9 pure germinomas, 2 mature teratomas, and 1 mixed germinoma- mature teratoma. Cases 13-18 could be categorized as members of the IPG and included 5 mixed GCTs and 1 immature teratoma. Cases 19-21 belonged to the PPG and included 3 mixed GCTs with YST component predominance (Additional file 1-B).

### The MicroRNA signatures associated with the different pediatric CNS GCT prognostic groups

Global miRNA expression patterns (the "miRNome") were analyzed in 12 cases (case 1-6, 12-14 & 16-18 in Additional file 1-B). Differentially expressed miRNAs that correlated with the germinoma group (GPG) and the NGMGCT group (IPG/PPG) were identified by 2-tailed Student's t-test with a significance level of *p *< 0.05 plus ≧2-fold changes. Their discrimination ability was assessed by principle component analysis (PCA). Thus, patients within the different prognosis groups were separated by their distinct miRNA profiles (Figure 1B). A heat map of these miRNAs indicates the unique expression levels associated with each prognostic group (Figure 1C). Two miRNAs (hsa-miR-142-5p and hsa-miR-146a) are enriched in the germinoma group (GPG) and 19 miRNAs are enriched in the NGMGCT group (IPG/PPG) (Figure 1C). The differential expression levels of the miRNAs across the two different histological categories and prognostic groups of the pediatric CNS GCTs were organized by array hybridization intensity (Figure 1D) and verified by quantitative PCR (qPCR) (Figure 1E). The expression levels of hsa-miR-142-5p, hsa-miR-335 and miR-654-3p were found to be different when the patients in these two different groups were compared (Figure 1D-E).

### Stem cell traits associated with the expression patterns of protein-coding gene within the NGMGCT group

**Figure 2:**
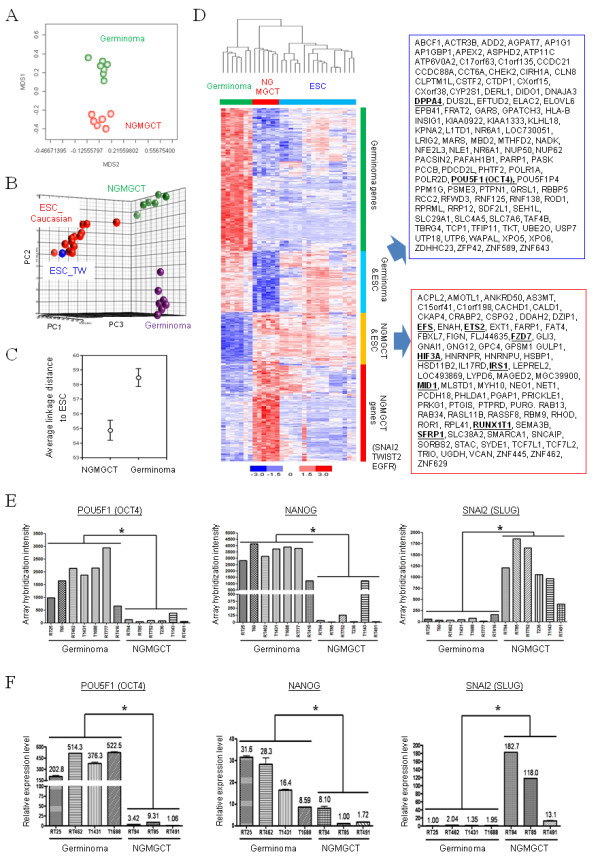
**Gene expression analysis of the different GCT subgroups**. (**A**) A multidimensional scaling (MDS) plot containing the differentially expressed genes (690 probe sets, *q *< 0.001). Each spot represents a single array. (**B**) A comparison of the transcriptome traits between ESCs and NGMGCTs by principal component analysis (PCA). (**C**) Relationships between ESCs, germinomas and NGMGCTs. Average linkage Euclidean distances between the tissues and ESC were calculated using genes distinguishing the filtrated 690-probe set. The confidence limits shown represent the standard error. (**D**) A heat map shows genes enriched in the ESCs and in the different prognostic groups (*q *< 0.001). (**E-F**) Real-time PCR validation of the microarray data. Mean expression levels of the examined genes were compared to that of the GAPDH control. Each bar represents a different individual (**F**). The genes' array hybridization signals are also shown (**E**).

The expression patterns of the protein-coding genes of the same 12 cases described above together with 1 additional germinoma case (case 7 in Additional file 1-B), were also analyzed. In total, 399 probe sets were specifically enriched in the germinoma group (GPG) compared to 292 ones in NGMGCT group (IPG/PPG) with a strict positive false discovery rate (pFDR) threshold of *q *< 0.001 (Additional file 2). The discrimination ability of these probe sets was assessed by a multidimensional scaling (MDS) assay (Figure 2A). The top 50 transcripts most strongly expressed in the germinoma group (GPG) or the NGMGCT group (IPG/PPG) among the pediatric CNS GCTs are shown in Table 1 and 2, respectively. In the germinoma group (GPG), the presence of MMP-12, which is involved in promoting tumor metastasis, needs to be noted [31] (Table 1, labeled by a asterisk). Podoplanin, a significant lymphatic endothelial cell marker, is also found in the top 50 genes of this group. Podoplanin is expressed by cancer associated fibroblasts (CAFs) and has been shown to be correlated with a poor prognosis in lung adenocarcinomas [32]. In addition, POU5F1 (alias OCT4), a significant transcription factor involved in maintaining the stemness of ES cells [33], is also among the top 50 genes in this group (Table 1, labeled by asterisks). Among the members of the GP group, the NANOG and KLF4 stemness factors are overexpressed (*q *< 0.01, data not shown). These stemness genes can induce pluripotency in somatic cells and then reprogram them back to a pluripotent status so that they have the essential characteristics of embryonic stem (ES) cells [33,34]. Another pluripotency associated gene, DPP4 (developmental pluripotency associated 4), is also highly expressed in germinomas. Finally, spermatogenesis- and oogenesis-related genes, such as SPATA2 (spermatogenesis associated 2), SPESP1 (sperm equatorial segment protein 1) and GTSF1 (gametocyte specific factor 1), were also found to be expressed more abundantly in germinomas than in NGMGCTs (Table 1).

**Table 1:** Top 50 known genes in TW germinomas.

Probe Set ID	UniGene ID	Gene Title	Gene Symbol	Location
207522_s_at	Hs.513870	ATPase; Ca++ transporting; ubiquitous	ATP2A3	chr17p13.3

1552487_a_at	Hs.459153	basonuclin 1	BNC1	chr15q25.2

219928_s_at	Hs.511983	calcium binding tyrosine-(Y)-phosphorylation regulated	CABYR	chr18q11.2

219578_s_at	Hs.547988	cytoplasmic polyadenylation element binding protein 1	CPEB1	chr15q25.2

1564491_at	Hs.590784	chromosome X open reading frame 18	CXorf18	chrXq27.2

206588_at	Hs.131179	deleted in azoospermia-like	DAZL	chr3p24.3

228057_at	Hs.480378	DNA-damage-inducible transcript 4-like	*DDIT4L	chr4q23

221630_s_at	Hs.223581	DEAD (Asp-Glu-Ala-Asp) box polypeptide 4	DDX4	chr5p15.2-p13.1

220004_at	Hs.125507	DEAD (Asp-Glu-Ala-Asp) box polypeptide 43	DDX43	chr6q12-q13

220493_at	Hs.98586	doublesex and mab-3 related transcription factor 1	DMRT1	chr9p24.3

232985_s_at	Hs.317659	developmental pluripotency associated 4	*DPPA4	chr3q13.13

210868_s_at	Hs.412939	ELOVL family member 6; elongation of long chain fatty acids	*ELOVL6	chr4q25

1555299_s_at	---	endogenous retroviral family W; env(C7); member 1 (syncytin)	ERVWE1	chr7q21-q22

1553614_a_at	Hs.652066	hypothetical protein FLJ25694	FLJ25694	chr13q21.31

207899_at	Hs.1454	gastric inhibitory polypeptide	GIP	chr17q21.3-q22

227711_at	Hs.524476	gametocyte specific factor 1	*GTSF1	chr12q13.2

219863_at	Hs.26663	hect domain and RLD 5	*HERC5	chr4q22.1

209398_at	Hs.7644	histone cluster 1; H1c	HIST1H1C	chr6p21.3

214455_at	Hs.553506	histone cluster 1; H2bc	HIST1H2BC	chr6p21.3

223861_at	Hs.298312	HORMA domain containing 1	HORMAD1	chr1q21.2

217522_at	Hs.660831	potassium channel; subfamily V; member 2	KCNV2	chr9p24.2

219955_at	Hs.685462	LINE-1 type transposase domain containing 1	L1TD1	chr1p31.3

220665_at	Hs.242183	leucine zipper protein 4	LUZP4	chrXq23

205668_at	Hs.153563	lymphocyte antigen 75	LY75	chr2q24

229475_at	Hs.651245	maelstrom homolog (Drosophila)	MAEL	chr1q24.1

207534_at	Hs.73021	melanoma antigen family B; 1	MAGEB1	chrXp21.3

206218_at	Hs.113824	melanoma antigen family B; 2	MAGEB2	chrXp21.3

214397_at	Hs.25674	methyl-CpG binding domain protein 2	MBD2	chr18q21

204580_at	Hs.1695	matrix metallopeptidase 12 (macrophage elastase)	*MMP12	chr11q22.3

204702_s_at	Hs.404741	nuclear factor (erythroid-derived 2)-like 3	NFE2L3	chr7p15-p14

229352_at	Hs.657932	sperm equatorial segment protein 1	*SPESP1	chr15q23

209626_s_at	Hs.520259	oxysterol binding protein-like 3	OSBPL3	chr7p15

204879_at	Hs.468675	podoplanin	*PDPN	chr1p36.21

210265_x_at	Hs.450254	POU class 5 homeobox 1 pseudogene 3	*POU5F1(OCT4)	chr12p13.31

225579_at	Hs.274415	PQ loop repeat containing 3	PQLC3	chr2p25.1

204086_at	Hs.30743	preferentially expressed antigen in melanoma	PRAME	chr22q11.22

218700_s_at	Hs.115325	RAB7; member RAS oncogene family-like 1	RAB7L1	chr1q32

1558668_s_at	Hs.351068	spermatogenesis associated 22	*SPATA22	chr17p13.3

223883_s_at	Hs.309767	serine/threonine kinase 31	STK31	chr7p15.3

1553599_a_at	Hs.506504	synaptonemal complex protein 3	SYCP3	chr12q

39318_at	Hs.2484	T-cell leukemia/lymphoma 1A	TCL1A	chr14q32.1

206413_s_at	Hs.510368	T-cell leukemia/lymphoma 1B	TCL1B	chr14q32.1

215356_at	Hs.646351	tudor domain containing 12	TDRD12	chr19q13.11

223530_at	Hs.144439	tudor and KH domain containing	TDRKH	chr1q21

227642_at	Hs.156471	Transcription factor CP2-like 1	TFCP2L1	chr2q14

228505_s_at	Hs.487510	transmembrane protein 170	TMEM170	chr16q23.1

208275_x_at	Hs.458406	undifferentiated embryonic cell transcription factor 1	UTF1	chr10q26

1553197_at	Hs.371738	WD repeat domain 21C	WDR21C	chr8q21.3

230193_at	Hs.709837	WD repeat domain 66	WDR66	chr12q24.31

243161_x_at	Hs.335787	zinc finger protein 42 homolog (mouse)	ZFP42	chr4q35.2

**Table 2:** Top 50 known genes in TW NGMGCTs.

Probe Set ID	UniGene ID	Gene Title	Gene Symbol	Location
219935_at	Hs.58324	ADAM metallopeptidase with thrombospondin type 1 motif; 5	ADAMTS5	chr21q21.3

219087_at	Hs.435655	asporin	ASPN	chr9q22

205433_at	Hs.420483	butyrylcholinesterase	BCHE	chr3q26.1-q26.2

236179_at	Hs.116471	cadherin 11; type 2; OB-cadherin (osteoblast)	*CDH11	chr16q22.1

212865_s_at	Hs.409662	collagen; type XIV; alpha 1	*COL14A1	chr8q23

202311_s_at	Hs.709197	collagen; type I; alpha 1	*COL1A1	chr17q21.33

229218_at	Hs.489142	collagen; type I; alpha 2	*COL1A2	chr7q22.1

208096_s_at	Hs.47629	collagen; type XXI; alpha 1	*COL21A1	chr6p12.3-p11.2

232458_at	Hs.443625	Collagen; type III; alpha 1 (Ehlers-Danlos syndrome type IV)	*COL3A1	chr2q31

212489_at	Hs.210283	collagen; type V; alpha 1	*COL5A1	chr9q34.2-q34.3

221729_at	Hs.445827	collagen; type V; alpha 2	*COL5A2	chr2q14-q32

202575_at	Hs.405662	cellular retinoic acid binding protein 2	CRABP2	chr1q21.3

204619_s_at	Hs.695930	chondroitin sulfate proteoglycan 2 (versican)	CSPG2	chr5q14.3

232090_at	Hs.584880	Dynamin 3	DNM3	chr1q24.3

204463_s_at	Hs.183713	endothelin receptor type A	EDNRA	chr4q31.23

204400_at	Hs.24587	embryonal Fyn-associated substrate	*EFS	chr14q11.2-q12

203184_at	Hs.519294	fibrillin 2 (congenital contractural arachnodactyly)	FBN2	chr5q23-q31

231130_at	Hs.645700	FK506 binding protein 7	FKBP7	chr2q31.2

204359_at	Hs.533710	fibronectin leucine rich transmembrane protein 2	FLRT2	chr14q24-q32

222853_at	Hs.41296	fibronectin leucine rich transmembrane protein 3	FLRT3	chr20p11

243278_at	Hs.656280	forkhead box P2	FOXP2	chr7q31

203706_s_at	Hs.173859	frizzled homolog 7 (Drosophila)	*FZD7	chr2q33

205498_at	Hs.125180	growth hormone receptor	GHR	chr5p13-p12

227070_at	Hs.631650	glycosyltransferase 8 domain containing 2	GLT8D2	chr12q

204237_at	Hs.470887	GULP; engulfment adaptor PTB domain containing 1	GULP1	chr2q32.3-q33

224997_x_at	Hs.533566	H19; imprinted maternally expressed transcript	H19	chr11p15.5

215446_s_at	Hs.102267	lysyl oxidase	LOX	chr5q23.2

204069_at	Hs.526754	Meis homeobox 1	MEIS1	chr2p14-p13

207480_s_at	Hs.510989	Meis homeobox 2	MEIS2	chr15q14

206201_s_at	Hs.170355	mesenchyme homeobox 2	MEOX2	chr7p22.1-p21.3

203637_s_at	Hs.27695	midline 1 (Opitz/BBB syndrome)	MID1	chrXp22

222722_at	Hs.708130	osteoglycin	OGN	chr9q22

213568_at	Hs.253247	odd-skipped related 2 (Drosophila)	OSR2	chr8q22.2

225975_at	Hs.591691	protocadherin 18	PCDH18	chr4q31

203131_at	Hs.74615	platelet-derived growth factor receptor; alpha polypeptide	PDGFRA	chr4q11-q13

212915_at	Hs.434900	PDZ domain containing RING finger 3	PDZRN3	chr3p13

227419_x_at	Hs.204947	placenta-specific 9	PLAC9	chr10q22.3

210809_s_at	Hs.136348	periostin; osteoblast specific factor	POSTN	chr13q13.3

238852_at	Hs.657841	Paired related homeobox 1	PRRX1	chr1q24

208131_s_at	Hs.302085	prostaglandin I2 (prostacyclin) synthase	PTGIS	chr20q13.13

214043_at	Hs.446083	protein tyrosine phosphatase; receptor type; D	PTPRD	chr9p23-p24.3

225946_at	Hs.696433	Ras association (RalGDS/AF-6) domain family member 8	RASSF8	chr12p12.3

232060_at	Hs.654491	receptor tyrosine kinase-like orphan receptor 1	ROR1	chr1p32-p31

205529_s_at	Hs.368431	runt-related transcription factor 1; translocated to; 1	RUNX1T1	chr8q22

202037_s_at	Hs.213424	secreted frizzled-related protein 1	*SFRP1	chr8p12-p11.1

213139_at	Hs.360174	snail homolog 2 (Drosophila)	*SNAI2 (SLUG)	chr8q11

228821_at	Hs.709275	ST6 beta-galactosamide alpha-2;6-sialyltranferase 2	ST6GAL2	chr2q11.2-q12.1

209651_at	Hs.513530	transforming growth factor beta 1 induced transcript 1	TGFB1I1	chr16p11.2

203083_at	Hs.371147	thrombospondin 2	THBS2	chr6q27

229404_at	Hs.708196	twist homolog 2 (Drosophila)	*TWIST2	chr2q37.3

In the NGMGCT group (IPG/PGG), genes involved in cell adhesion and migration, such as cadherin 11 (CDH11) and various collagens, are abundantly expressed (Table 2, labeled by asterisks). SNAI2 (alias SLUG) and TWIST2, two key regulators involved in neural crest development and epithelial-mesenchymal transition (EMT), are also highly expressed in this group; these proteins are known to contribute heavily to cell motility and tumor metastasis [35]. Finally, genes such as FZD7 and SFRP1, which are involved in the Wnt signaling pathway, are also highly expressed (Table 2).

It has been recognized that aggressive and poor prognostic glioblastomas, as well as other tumors, acquire characters reminiscent of embryonic stem cells (ESCs) and the degree of ESC gene expression correlates with patient prognosis [36]. It is possible that pediatric CNS GCTs, especially the poor prognosis NGMCGTs, are reminiscent of ES cells. We compared the gene expression patterns of pediatric GCTs to those of Caucasian and Taiwanese ESC lines. PCA analysis showed that NGMGCTs have a closer relationship to ES cells (Figure 2B). The ESC array data from five different data sets (GSE7234, GSE7896, GSE9440 (for the Taiwanese ESC lines) and GSE9832 and GSE13828 (for the Caucasian ESC lines) were all grouped together (Figure. 2B) and possible batch effects during array analysis were ignored. To provide quantitative insights, we calculated the relationships between the GCT subgroups and the ESCs by measuring the average linkage Euclidean distances between them. NGMGCTs were found to closer to the ESC than the germinomas (Figure. 2C).

The closer relationship between NGMGCTs and ESCs was verified further by hierarchical clustering. As shown in Figure. 2D, clearly the NGMGCTs and ESCs form one group while the germinomas form another. In total, 100 genes commonly show high-expression between NGMGCTs and ESCs (Figure. 2D). Among these genes the following are notable. IRS1 (Insulin receptor substrate 1) is an effector of sonic hedgehog mitogenic signaling in cerebellar neural precursors [37] and regulates murine embryonic stem cell self-renewal [38] (Figure. 2D, underlined and in bold). MID1 is a RING finger transcription factor involved in Opitz syndrome and is expressed strongly in undifferentiated cells in the central nervous system as well as the gastrointestinal and respiratory tract epithelium of human embryos [39]. Embryonic oncogenes such as NET1 (neuroepithelial cell transforming gene 1), HIF3A (hypoxia inducible factor 3, alpha subunit), ETS2, RUNX1T1, and the Wnt signaling pathway genes (FZD7 and SFRP1) also appear in this cluster (Figure. 2D). However, notably, two key EMT genes, SNAI2 (SLUG) and TWIST2, are uniquely expressed by NGMGCTs (Figure. 2D).

Among the genes commonly found to show abundant expression in both the ESCs and germinomas, the pluripotent stemness genes DPP4 and POU5F1 (OCT4) are significant (Figure. 2D, underlined and in bold). The array hybridization signal for POU5F1 is shown in Figure. 2E. The high expression of POU5F1, as well as that of another stemness gene NANOG in germinomas, was verified by qPCR (Figure 2F). In contrast, SNAI2 (SLUG) is overexpressed in NGMGCTs (Figure 2E-F).

### Relationships between abundant microRNAs and their target mRNAs

The most differentiating miRNAs between the histological subgroups were used to predict their mRNA targets. This was performed by examining whether there were any candidate miRNA target genes, the expression of which became significantly higher in a given group of tumors, which also showed a correlated reduction in the related miRNAs. This analysis yielded miRNA-target pairs that showed opposite expression patterns in the same prognostic group (Table 3). In the germinoma group, the expression levels of RUNX1T1 and THRB were inversely correlated with expression of miR-146a, and the levels of NRP1, SVIL and PDGFRA were inversely correlated with the expression of miR-142-5p. Furthermore, RUNX1T1 is a putative target of both miR-142-5p and miR-146a (Table 3, underlined). In the NGMGCT group, inverse correlation expressions were also found between miRNAs and their candidate downstream targets (Table 3), specifically, miR-218, which is an intragenic miRNA of the overexpressed SLIT2 gene (Table 3, labeled by an asterisk).

**Table 3:** Signature miRNAs and their predicted targets in the opposite prognostic group.

miRNA	Mapping	Intragenic	Predicted Targets	*p *value
142-5p	17q22		ADAMTS5, BCHE, DCHS1, FIGN, FLJ10357, FLRT2, FZD7, HDAC4, MEIS2, NRP1, PDGFRA, PTPRD, RUNX1T1, SGCD, SVIL	2.09E-10

146a	5q33.3		C5orf23, PTGFRN, RPESP, RUNX1T1, SRR, THRB	5.89E-07

let-7e	19q13.43		*(NA)*	*(NA)*

26a	3p22.2	CTDSPL	ATP11C, C7orf42, CREBL2, DNAJA2, FRAT2, NFE2L3, **NUP50**, TFAP2C, **WAPAL**, ZNF655	4.23E-07

133b	6p12.2		*(NA)*	*(NA)*

181c	19p13.12		C17orf63, EPB41, NMT1	1.18E-03

154	14q32.2		*(NA)*	*(NA)*

218	4p15.31	*SLIT2	**NUP50**, SFMBT1, WDR66, WNT2B, ZDHHC23, ZNF313	1.82E-02

335	7q32.2		N4BP1, PHTF2, SLC45A3, **WAPAL**	3.34E-04

376a	14q32.31		BNC1, MAN1C1	1.45E-02

410	14q32.31		AGPAT7, DLG3, NMT1, OSBPL3, RGS16, ROD1, ZNRF2	2.27E-04

433	14q32.2	RTL1	PCCB, **WAPAL**	4.50E-02

503	Xq26.3		CREBL2, DNAJA2, KIAA1333, MBP, N4BP1, **NUP50**, PAFAH1B1, RNF138, WNT2B	1.41E-07

539	14q32.2		CCDC88A, FRAT2, LBA1, MYCL1, PSME3, SNAP29, **WAPAL**, XPO6	4.75E-05

543	14q32.31		ARFGEF2, ATP11C, CSNK1D, CTF8, OSBPL3, PAFAH1B1, PPTC7, RNF138, SDF2L1	2.38E-05

NA: No miRNA target could be found in the opposite group.

Underlined: Genes targeted by 2 microRNAs.

In bold and underlined: Genes targeted by more then 2 microRNAs.

The signature miRNAs in the same GCT prognosis group were found to target the same mRNAs. miR-503 and miR-543 both target PAFAH1B1 and RNF138, while miR-26a and miR-503 both target CREBL2 and DNAJA2 (Table 3, underlined). In addition, FRAT2 is a putative target of both miR-26a and miR-539, ATP11C is a target of both miR-26a and miR-543, NMT1 is a target of both miR-181c and miR-401, WNT2B is a target of both miR-218 and miR-503, N4BP1 is a target of both miR-335 and miR-503, and OSBPL3 is a target of both miR-410 and miR-543 (Table 3, underlined). Some mRNAs are even targeted by more than two miRNAs: NUP50 is targeted by three miRNAs (miR-26a, miR-218 and miR-503), while WAPAL is a target of four miRNAs (miR-26a, miR-335, miR-433 and miR-539 (Table 3, in bold and underlined, respectively). Thus it would seem that there are complex and highly interactive miRNA-mRNA genetic networks active in germinomas and NGMGCTs.

### Functional module and pathway analysis as a framework for the interpretation of GCT biology

**Figure 3:**
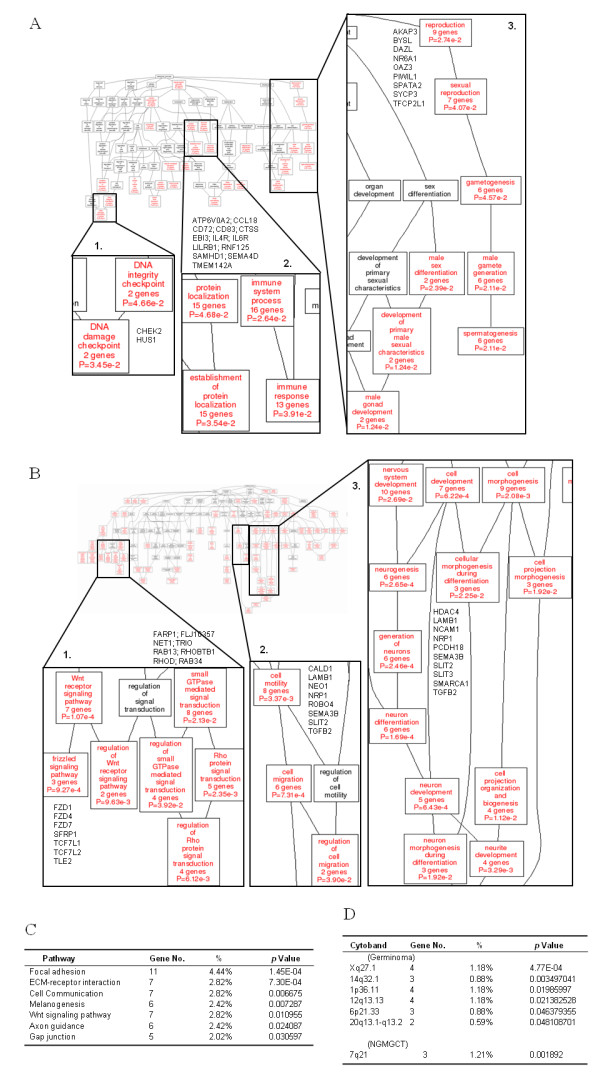
**Altered functional modules in the different pediatric GCT prognostic groups**. (**A-B**) Gene set enrichment analysis according to the Gene Ontology (GO) classification. Probe sets differentiating good prognostic CNS GCTs from intermediate/poor prognostic CNS GCTs were subjected to the GO database search via the DAVID 2008 interface. The number of genes, gene symbols, their percentages and the *p *values for each category that show significance (*p *< 0.05) and are enriched in either the good (**A**) or the intermediate/poor (**B**) prognostic group are listed. (**C**) KEGG pathways significantly enriched in the TW NGMGCT genes. The number of genes, their percentages in terms of total genes, and the *p *values for pathways that are significantly over-represented (*p *< 0.05 by the DAVID 2008 tool) are listed. (**D**) Distribution of signature genes on the chromosome cytobands.

The gene list outlined above gave us preliminary insights into the functional consequences of detected differential gene expression. To understand more about how the gene expression profiles might be correlated with pathogenesis and the various clinical phenotypes as well as to provide quantitative evidence, the signature mRNAs were subjected to a Gene Ontology (GO) database search [40] in order to find statistically overrepresented functional groups within the gene lists. The WebGestalt web tool [41] was applied to provide statistical analysis and visual presentation of the results. The GO categories of biological processes that were statistically overrepresented (*p *< 0.05) among genes of the germinoma group are shown in Figure 3A. Genes CHEK2 and HUS1, which are involved in the DNA damage checkpoint, were significantly overexpressed in germinomas (*p *= 3.45*10e-2; Figure 3A, panel 1). Another significant biological process associated with this group is related to the immune system processes (*p *= 2.64*10e-2; Figure 3A, panel 2, where the involved immune response genes are shown). Other predominant processes in the GP group include genes pertaining to reproduction (*p *= 2.74*10e-2) and male gonad development (*p *= 1.24*10e-2; Figure 3A, panel 3).

In contrast, the principal functions of the p-regulated genes in the NGMGCT group (IPG/PPG) of pediatric GCTs include those related to small GTPase (Rho protein especially) mediated signal transduction (Figure 3B, panel 1), cell motility (Figure 3B, panel 2) and various genes associated with active differentiation processes, in particular neuron differentiation (Figure 3B, panel 3). Seven genes involved in the Wnt receptor signaling pathway are also significantly active in this group (*p *= 1.07*10e-4; Figure 3B, panel 1). When the genes (*q *< 0.001) are subjected to a KEGG pathway database to obtain a similar module analysis using the DAVID 2008 web-based tool, the top-ranked canonical pathways in the NGMGCT group again include cell motility (such as Focal adhesion, ECM-receptor interaction and Gap junction), axon guidance and Wnt signaling (Figure 3C). Expression of Wnt pathway genes (such as FZDI, FZD3, FZD4, FZD5, FZD6 and SFRP1, SFRP2, FRZB, SFRP4) have been previously reported in a pluripotent human embryonal carcinoma cell line and in an embryonic stem cell [15], which supports the reliability of our functional module analysis. FZDI, FZD4, FZD7 and SFRP1 are also in our gene list (Table 2 and Additional file 2). The detailed locations of the signature genes are indicated in Additional file 3 and Additional file 4.

### Chromosome locations of the differentially expressed genes and cytogenetic analysis of the GCTs

Gene set enrichment analysis (GSEA) was performed by DAVID for all chromosomal arms using the entire gene list. NGMGCTs were found to shows significantly transcript expression in the 7q21 cytoband region, which contains 3 NGMGCT genes: GNG11 (guanine nucleotide binding protein (G protein), gamma 11), GNAI1 (G protein alpha inhibiting activity polypeptide 1) and FZD1 (frizzled homolog 1). In germinomas, genes were overexpressed at Xq27.1, 14q32.1 (TCL1A & 1B), 1p36.11 (CCDC21, ZNF593, FAM46B and C1orf135), 12q13.13, 6p21.33 (ABCF1, HIST1H2BK and C6orf136) and 20q13.1-q13.2 (Figure 3D). The POU5F1 (OCT4) germinoma gene, as well as SLC4A8, LOC57228 and C12orf44, are overexpressed at chr12q13.13. The spermatogenesis associated gene SPATA2, as well as PTPN1, are overexpressed at 20q13.1-q13.2 (Figure 3D).

**Figure 4:**
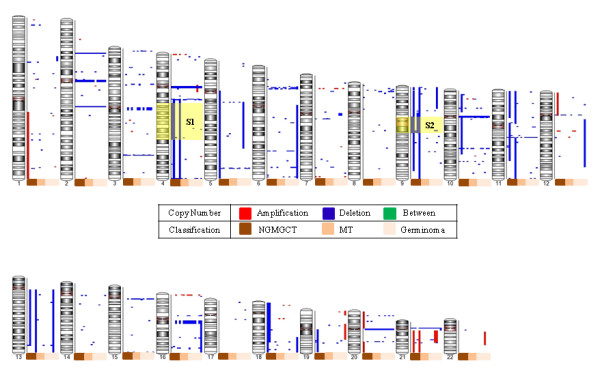
**Chromosomal aberrations in the TW germinomas, mature teratomas (MTs) and NGMGCTs**. The red bars on the right side of the chromosome idiograms indicate gain in these chromosomal regions, while blue bars indicate chromosomal loss. Two common copy number variation (CNV) regions (S1 & S2) in 3 out of 5 NGMGCT cases are highlighted.

It is likely that gene expression changes are attributable to underlying chromosomal aberrations. To identify such a correlation, we examined the cytogenetic abnormalities present in each GCT prognosis subtype. Copy number variation (CNV) analysis was performed on 15 pediatric CNS GCT cases (7 pure germinomas, 3 pure mature teratomas and 5 NGMGCTs; Additional file 1-B) in order to detect chromosomal aberrations. A data set containing 125 Human 1 M HapMap samples (generated by the Partek Inc.) was used as a copy number baseline. The aberrant chromosome regions in each tested individual are summarized in Additional file 5. As shown in Figure. 4, 3 out of 5 NGMGCT cases have a reduced DNA copy number between 4q13.3-4q28.3 (S1) and 9p11.2-9q13 (S2). The protein-coding genes and miRNAs located in these changed regions are shown in Table 4. BANK1, CXCL9, CXCL11, DDIT4L, ELOVL6 and HERC5 are within 4q13.3-4q28.3 and are relatively more abundant in germinomas (Table 4 and Additional file 2). DDIT4L, ELOVL6 and HERC5 are also among the top 50 most dominant genes in germinomas (Table 1).

**Table 4:** Deleted chromosomal regions in NGMGCTs and the genes within those regions.

	Cytoband	Start Nucleotide#	End Nucleotide#	Protein-coding genes	microRNAs
S1	4q13.3-4q28.3	75084501	131387610	ABCG2, ADAD1, ADH1A, ADH1B, ADH1C, ADH4, ADH5ADH6, ADH7, AFF1, AGPAT9, AGXT2L1, AIMP1, ALPK1, ANK2, ANKRD50, ANKRD56, ANTXR2, ANXA3, ANXA5, AP1AR, ARD1B, AREG, ARHGAP24, ARSJ, ART3, ATOH1, BANK1, BBS7, BBS12, BDH2, BMP2K, BMP3, BMPR1B, BTC, C4orf3, C4orf11, C4orf12, C4ord17, C4orf21, C4ord22, C4orf26, C4orf29, C4orf31, C4ord32, C4orf33, C4orf36, C4orf37, CAMK2D, CAPSP6, CCDC109B, CCDC158, CCNA2, CCNG2, CCN1, CDKL2, CDS1, CENPE, CEP170L, CFI, CISD2, CNOT6L, COL25A1, COPS4, COQ2, CXCL2, CXCL3, CXCL9, CXCL10, CXCL11, CXCL13, CXXC4, CTP2U1, DAPP1, DDIT4L, DKK2, DMP1, DNAJB14, DSPP, EEF1AL7, EGF, EIF4E, ELOVL6, EMCN, ENOPH1, ENPEP, EPGN, EREG, EXOSC9, FABP2, FAM13A, FAM13AOS, FAM47E, FAM175A, FAM190A, FAT4, FGF2, FGF5, FLJ20184, FRAS1, G3BP2, GAR1, GDEP, GK2, GPRIN3, GRID2, GSTCD, H2AAFZ, HADH, HELQ, HERC3, HERC5, HERC6, HNRPDL, HPGDS, HPSE, HSD17B11, HSD17B13, HSPA4L, IBSP, IL2, IL21, INTS12, INTU, KIAA1109, KLHL8, LARP1B, LARP7, LEF1, LIN54, LOC100192379, LOC256880, LOC285419, LOC285456, LOC641518, LOC729338, LRIT3, MAD2L1, MANBA, MAPK10, MAPKSP1, MEPE, METAP1, METTL14, MFSD8, MMRN1, MRPL1, MRPS18C, MTHFD2L, MTTP, MYOZ2, NAAA, NAP1L5, NDST3, NDST4, NEUROG2, NFKB1, NHEDC1, NHEDC2, NKX6-1, NPNT, NUDT6, NUDT9, NUP54, OSTC, PAPSS1, PAQP3, PARM1, PCNAP1, PDE5A, PDHA2, PDLIM5, PGRMC2, PHF17, PIGY, PITX2, PKD2, PLA2G12A, PLAC8, PLK4, PPA2, PPBPL2, PPEF2, PPM1K, PPP3CA, PRDM5, PRDM8, PRKG2, PRSS12, PTPN13, QRFPR, RAP1GDS1, RASGEF1B, RCHY1, RG9MTD2, RPL34, RRH, SCARB2, SCD5, SCLT1, SDAD1, SEC24B, SEC24D, SEC31A, SEP11, SGMS2, SHROOM3, SLC10A6, SLC25A31, SLC39A8, SMARCAD1, SNCA, SNHG8, SNORA24, SPARCL1, SPATA5, SPP1, SPRY1, *SYNPO2, TACR3, TBCK, TET2, THAP6, THAP9, TIFA, TIGD2, TMEM150C, TMEM155, TMSL3, TNIP3, TRAM1L1, TRPC3, TSPAN5, UBE2D3, YGT8, UNC5C, USO1, USP53, WDFY3	hsa-miR-302A, has-miR-302B, has-miR-302C, has-miR-302D, has-miR-367, has-miR-575, has-miR-577

S2	9p11.2-9q13	44703105	70128535	ANKRE20A2, ANKRD20A3, ANKRD20A4, AQP7P1, AQP7P2, CBWD3, CBWD5, CBWD6, CCDC29, FAM27A, FAM27B, FAM27C, FAM74A4, FAM75A5, FAM75A7, FXOD4L2, FOXD4L3, FOXD4L4, FOXD4L5, FOXD4L6, KGFLP1, LOC100133920, LOC440839, LOC440896, LOC442421, MGC21881, PGM5P2	(No know ones)

Underlined and in bold: Genes which are relatively more dominant in germinomas.

Asterisk: Discussed in the text.

## Discussion and Conclusions

GCT is a specific type of CNS tumor with several subtypes. The two major forms of these tumors, germinoma (GPG) and NGMGCT (IPG/PPG), present with different clinical behaviors, differences in sensitivity to therapeutic regimens and different outcomes. The overall survival of patients with germinomas is significantly better than that of patients with NGMGCTs in our series (Figure 1A) and this is similar to other previously reported series [42,43]. To explore the molecular difference between these two different histological/therapeutic prognostic groups, we have identified with confidence a number of differentially expressed miRNAs and mRNA; these permit an interpretation of the clinical survival variations and downstream hypothesis testing. The various divergent biological functions that correlate with the clinical observations are also revealed.

Among these miRNAs, miR-142-5p and miR-146a are upregulated in the pediatric germinomas (GP group) when compared to the NGMGCTs (IPG/PPG). Up to the present, no miRNA profile of pediatric GCTs has been published. A miRNome report on adult gonadal GCTs showed that, for each GCT subtype, the miRNA patterns are quite different [24]. In their dataset, miR-142-5p and miR-146a are also more abundant in adult seminomas than in gonadal ECs [24]. In addition, let-7e, miR-133b, miR-218 and miR-654-3p are also abundant in both pediatric NGMGCTs and adult ECs (Figure 1C) [24]. However, the notable discrepancies are miR-181c and miR-218, the expression levels of which are more abundant in adult testicular seminomas but are lower in pediatric intracranial germinomas (Figure 1) [24]. The unique expression pattern of these miRNAs in pediatric CNS GCTs may reflect the differences in pathogenesis mechanisms between adult and pediatric GCTs [17], or, alternatively, the variation in genetic makeup between Western and Taiwanese patients.

We also correlated the transcript levels of miRNAs to their candidate targets in order to identify microRNA-mRNA target pairs (Table 3). It has been shown that some miRNAs, such as miR-1, can downregulate the transcript levels of a large number of target genes in mammalian cells [18]. Two large scale proteomic studies published very recently have shown that, although some microRNA target proteins are repressed without detectable changes in mRNA levels, more than a third of translational repressed targets also display detectable mRNA destabilization and, for the more highly repressed targets, mRNA destabilization usually makes up the major component of repression [19,20]. Gene expression microarrays can therefore be, and have been, applied for the identification of downstream targets for miRNAs [44-46]. However, proof of direct binding between those miRNAs and target mRNAs, as well as the direct translational repression of target mRNAs, is still needed. Such confirmation will require more wetlab experiments such as immunoblotting and reporter assays.

When compared with NGMGCTs, the germinomas largely recapitulate the features of self-renewing pluripotent human embryonic stem (hES) cells, such as involvement of POU5F1 (OCT4), NANOG and KLF4 (*q *< 0.01). Both seminomas and embryonal carcinomas are known to express stem cell markers, such as POU5F1 and NANOG [47,48]. In addition, in an attempt to find coordinated overexpressed gene clusters in GCTs, Korkola *et al*. found NANOG at chromosome 12p13.31 is overexpressed in undifferentiated (embryonal carcinomas and seminomas) tumors versus differentiated (teratoma, yolk sac tumor, and choriocarcinoma) tumors [16]. By overexpressing POU5F1, NANOG and KLF4, it is now possible to reprogram the transcriptomes of somatic primary cells, which results in their dedifferentiation from matured cells to ES cell-like iPS (induced pluripotent stem) cells [49]. The abundant expression of these dedifferentiation factors in germinomas therefore mirrors the more undifferentiated histopathological characteristics of these tumors. Whereas such similarities have previously been described for adult and pediatric seminomas [16,17,47,48], we now know that this also applies to Asian pediatric CNS germinomas.

Although germinomas abundantly express the above three stemness factors, it is NGMGCTs (IPG/PPG) who show a closer gene expression pattern to ESCs (Figure 2C). This observation is consistent with pervious global gene expression reports whereby the gene expression patterns of human ES cell lines are similar to those of the human embryonal carcinoma cell samples but are more distantly related to those of seminoma samples [12]. The close relationship between NGMGCTs and ES cells supports the hypothesis that germinomas are closely related to primordial germ cells (PGCs), and EC cells/NGMGCTs represent a reversion to a more ICM- or primitive ectoderm-like cell type [12]. Whether germinomas and zygotes/blastomeres share similar mRNA or microRNA profiles is under investigation at present. The close relationship between NGMGCTs and ES cells may additionally be reflected in the worse prognosis for these tumors. Recently, via novel genomic approaches, it has been shown that aggressive and poor prognostic tumors, such as glioblastomas, inherit preferential ES cell gene expression profiles [36]. The similarity between pediatric NGMGCTs and human ES cells may therefore reflect the clinical observation that CNS NGMGCTs are more malignant and show a higher fatality rate than germinomas.

The close relationship in genetic makeup between NGMGCTs and ESCs also suggest that factors other than POU5F1 (OCT4), NANOG or KLF4 are responsible for ESC gene expression. In this study, we found that two key epithelial-mesenchymal transition (EMT) regulators, SNAI2 (SLUG) and TWIST2, are abundantly expressed in the NGMGCT group (IPG/PPG) (Table 2 and Figure 2D). It has been reported that EMT transcription factors, SNAI1 (alias SNAIL) and TWIST, can independently dedifferentiate mammalian cancer cells and induces the generation of cancer stem-like cells, which then form mammospheres [50]. It is possible that SNAI2 (SLUG) and TWIST2 behaves like Snail and TWIST and can introduce malignancy and stemness in pediatric GCTs. Targeting oncogenic stemness genes or EMT-related embryonic signaling pathways (such as the Wnt pathway, Figures 2D &3C) may differentiate a highly malignant NGMGCT into a more matured transcriptome type, thereby increasing the sensitivity of these tumors to the classical therapeutic regimen of radical resection, irradiation and chemotherapy, which would produce a better prognosis for the patients.

In addition to stemness genes (such as genes involved in reproduction and male gonad development), the germinomas were found to overexpress genes involved in the DNA damage checkpoint, which indicates active DNA integrity checking in the germinomas and thereby reflects why the clinical phenotype of germinomas has a better prognosis (Figure 3A). Among the other genes that were found to be expressed abundantly in germinomatous tissues were genes associated with the immune system process and this correlates with the abundant lymphocytic infiltration of germinomas found during histological observation. Relative to germinomas, we observed a significant enrichment of overexpression of differentiation and morphogenesis (especially neurogenesis) genes in NGMGCTs, which correlates with the differentiated state of these tumor cells (Figure 3B). There is also evidence of overexpression of genes in the Wnt/β-catenin pathway in our dataset (Figures 3B-C), which is consistent with previous studies of nonseminomatous malignant GCTs [15,51]. In concordance with the higher recurrence and disseminating clinical behaviors of NGMGCTs, a significant enrichment for overexpression of motility, tight junction, focal adhesion, and adherent junction genes in NGMGCTs was observed (Figures 3B-C). Our results thereby integrate molecular profiles with clinical observations and provide a better understanding of the underlying molecular mechanisms. The combined targeting of hub genes involved in all these biological modules by a cocktail therapy-like regimen may eventually lead to an alleviation of these malignant CNS tumors.

During the submission of this manuscript, a very recent reference based on testis GCTs identified gene expression signatures that predicted outcomes in patients with extra-cranial adult GCTs [52]. We compared the age and tumor characteristics between our series against the genomic study group of CNS GCTs in children and the reported study of extra CNS GCTs in adult men (Additional file 1-C) [52]. In our series and the genomic study of CNS GCTs, both germinomas and NGGCTs in children younger than 18 years old were included, whereas Korkola's study involved adult men with nonseminomatous GCTs (NSGCTs) [52]. In our series, 118 tumors were pure germinomas or tumors with a germinoma component, 49 tumors were pure teratoma or tumors with a teratoma component, and 27 cases were classified as YSTs including 10 pure YSTs, 11 tumors with a YST component, and 6 cases with serum AFP elevation (pure immature teratomas excluded). Among the 21 cases with genomic studies, 9 tumors were pure germinomas, 2 tumors were pure mature teratomas, and 9 tumors were mixed GCTs, including one mature teratoma with serum AFP elevation and one germinoma with serum AFP elevation. The correlation of tumor characteristics between the studies of Korkola *et al*. and ours in Additional file 1-C constituted the basis for the comparison of genomic molecular findings across the different therapeutic prognostic groups and histology between these two studies.

Korkola *et al*. concluded that using a 140-gene signature, they could predict 5-year overall survival (OS) (*p *< 0.001) [52]. Both our study and that of Korkola *et al*. identified good outcome GCTs express gene sets involved in immune function and the repression of differentiation (such as POU5F1/OCT4), while poor outcome GCTs express genes involved in active differentiation (in particular, neuron differentiation) (Fig. 3) [52]. A 10-gene prognosis model was also built using a univariate Cox model. When the samples were dichotomized by median score, there was significant separation of the survival curves (*p *< 0.002) [52]. These 10 genes were STX6, CFLAR, FNBP1, ITSN2, SYNE1, MAP3K5, PTGDS, PXMP2, IRAK4, and RABGAP1L [52]. Among these 10 genes STX6 (syntaxin 6) and CFLAR (CASP8 and FADD-like apoptosis regulator) are over-expressed in our germinoma group (*q *< 0.01). It will be interesting to fit their prognosis signatures onto our dataset to see whether GCTs of different anatomic locations, ages and ethnic populations express similar prognosis genes. However, since all the tissues used in our study were freshly collected over the last 2 years, only one death has been recorded so far (Additional file 1). As a result, this work needs to be carried out at a later stage.

The variation in chromosome copy number variation (CNV) regions between germinomas and NGMGCTs were mapped to cytobands 4q13.3-4q28.3 and 9p11.2-9q13 (Figure 4). Chromosome abnormality analysis of adult testicular germ cell tumors (tGCTs) revealed that all GCTs show 12p gain [25,26]. In 2007, Palmer *et al*. used metaphase-based comparative genomic hybridization (CGH) to analyze genomic imbalance in 34 pediatric GCTs (22 yolk sac tumors (YSTs), 11 germinomatous tumors and one metastatic embryonal carcinoma). The YSTs showed an increased frequency of 1p loss (*p *= 0.003), 3p gain (*p *= 0.02), 4q loss (*p *= 0.07) and 6q loss (*p *= 0.004) compared to germinomas [30]. Most of their cases were from the testis, the ovary or the sacrococcygeal region and only 2 germinomas and 1 YST brain GCTs were included [30]; this is a possible explanation of the discrepancies between their results and ours. We also observed 4q loss in the NGMGCTs (including YSTs), suggesting that genomic imbalance in this region, and the genes/miRNAs encoded by this chromosomal region, may play a crucial tumor suppressing role during NGMGCT pathogenesis and affect clinical performance (Table 4). Six genes (BANK1, CXCL9, CXCL11, DDIT4L, ELOVL6 and HERC5) within 4q13.3-4q28.3 showed higher expression levels in the germinomas (Table 4). DDIT4L, ELOVL6 and HERC5 are among the top 50 highly expressed genes in germinomas (Table 1). A putative GCT tumor suppressor gene SYNPO2 (Synaptopodin 2), also known as myopodin, is also within the 4q13.3-4q28.3 deletion region (Table 4). SYNPO2 has recently been shown to have the highest predictive value when assessing 5-year overall survival [52], which is consistent with a possible role as a tumor suppressor. However, we do not observe differential SYNPO2 expression between NGMGCTs and germinomas (Table 4). It is unclear whether SYNPO2 expression is also downregulated in Taiwanese germinomas compared to normal brains. In addition, whether survival predictors derived from Western cases can be applied to Asian patients still awaits elucidation.

Recently two independent genome-wide association studies (GWAS) have reported on susceptibility loci associated with tGCT: Kanetsky *et al*. mapped seven markers at 12p22 near KITLG (c-KIT ligand) and two markers at 5q31.3 near SPRY4 (sprouty 4) [53]; furthermore Rapley *et al*. identified loci on chromosome 5, 6 and 12 [54]. A third locus, in an intron of BAK1, a gene that promotes apoptosis, was also identified by Rapley *et al*. [54]. Similarly, the CGH profiles in childhood GCTs have been reported to resemble those in adults [55,56]. In terms of cytogenetics differences between the different histological entities, loss of chromosome 19 and 22 material and gain of 5q14-q23, 6q21-q24 and 13q material were found to occur at a significantly lower frequency in seminoma adult tGCTs compared to non-seminoma adult tGCTs [25]. Among Taiwanese pediatric GCTs, no common copy number variation (CNV) could be found in either the germinomas or the mature teratomas (Figure 4). The divergence between our results and published Caucasian ones may be partly due to the different ethnic samples used, the application of different bioinformatics algorithms and the fact that we compared the differences between germinomas and NGMGCTs but not common aberrations across all GCTs.

In summary we have identified miRNome, mRNA signatures and CNV regions that are associated with two pediatric GCT histological entities (germinoma and NGMGCTs) and two prognostic groups (GPG and IPG/PPG). The clinical discrepancies between the two histological entities (germinomas of GPG and NGMGCTs of IPG/PPG) are therefore mirrored by their differences in global transcriptome patterns and their unique stem cell traits. One of the interesting questions that remain is whether pediatric GCTs from other ethnic background also express similar transcriptome traits and CNV regions. If Caucasian and Taiwanese GCTs possess unique transcriptome traits, therapeutic and diagnostic experience from Western countries may not be applicable directly to Asian or Taiwanese patients. Therefore, the genes and miRNAs identified here hold the potential of being novel therapeutic targets and may be used for further differentiation therapy. The Wnt pathway, for example, is activated in NGMGCTs (Figure 3C), and drugs targeting this specific pathway may hold potential as a treatment approach to NGMGCTs. Transdifferentiating ESC-like NGMGCTs into a benign status may also be a novel and useful tactic against these fatal pediatric tumors.

## Methods

### Patient details and microarray expression data

All procedures were approved by the Institutional Review Board of the Taipei Veteran General Hospital, Taiwan and informed consent was obtained from each subject or the subject's guardian according to the Helsinki Declaration. In this study, we reviewed a clinical database containing 176 cases of primary pediatric CNS GCTs involving patients less than 18 years old; the database was collected from 1970 to 2007 at Taipei Veterans General Hospital (Taipei VGH). Among them, RNA samples from the hospital tissue bank were obtained in 13 cases, and mRNA and miRNA microarray analysis were performed in 13 cases and 12 cases respectively. The histological types of this series of 176 primary CNS GCTs and other selected clinical data are summarized in Additional file 1-A. Excluding operative mortality, the overall survival rates of the 95 germinoma cases and 59 NGMGCT cases that form this series were studied to support the difference in malignancy and outcome between these two groups of CNS GCTs. Overall survival was analyzed by the Kaplan-Meier method, and the log-rank test was applied to compare the cumulative survival durations in the different patient groups and this was done using SPSS statistics software (SPSS Inc., Chicago, Illinois, USA).

The clinical features of the 22 CNS GCT cases used in microarray studies are listed in Additional file 1-B in order to help correlation with the results of the genomic analysis. In the transcriptome analysis, 13 cases had both mRNA and miRNA analyzed, except that case 7 had only mRNA analyzed (Additional file 1-B); the latter was due to insufficient RNA being available. The histological subtypes in the dataset are germinoma (6), mixed GCT of germinoma and mature teratoma (1), immature teratoma (1), mixed GCTs of NGMGCTs category (4), YST (1). Caucasian embryonic stem cell (ESC) array data that had been previously published [57], and the array data of Taiwanese ESC line hES-T3 (T3ES) were downloaded from the Gene Expression Omnibus (GEO; http://www.ncbi.nlm.nih.gov/geo/) database (accession number GSE9440) [58]. All ESC and GCT mRNA array data were implemented using the Affymetrix Human Genome U133 Plus 2.0 chips. The ESC array dataset was downloaded from GEO datasets GSE7234, GSE7896, GSE9440 (for Taiwanese ESC lines) together with GSE9832 and GSE13828. All GCT raw array data (including gene expression array, microRNA array and SNP array) are available from the GEO database (accession number GSE19350).

### MicroRNA microarray and data analysis

The Agilent Human miRNA Microarray Kit V2 (Agilent, Foster City, CA, USA) containing probes for 723 human microRNAs from the Sanger database v10.1 was used. GeneSpring GX 9 software (Agilent, USA) was used for value extraction. A 2-tailed Student's t-test was then used for the calculation of the *p *value for each miRNA probe. Principal component analysis (PCA) was performed using the Partek Genomics Suite software http://www.partek.com to provide a visual impression of how the various sample groups are related. To predict the downstream mRNA targets of the miRNAs, the TargetScan web tool http://genes.mit.edu/targetscan/index.html was used. The miRNA-target pairs were then mapped by examining whether there were any candidate miRNA target genes whose expressions became diminished in a given group of tumors while there was overexpression of the correlated miRNAs. A Fisher's exact test was used to examine whether the associations obtained were by chance or not.

### Copy number variation (CNV)

The materials used in the CNV study were fresh frozen tumor tissues, and the genomic DNA from each sample was isolated using a DNeasy Blood & Tissue Kit according to the manufacturer's instructions (Qiagen, GmbH, Germany). The Human610-Quad Beadchip (Illumina Technologies, USA) with 550,000 selected tag SNPs and 60,000 genetic markers covering 4.7 KB mean probe spatial resolution was used for the analysis. Normalized bead intensity data obtained for each sample were loaded into the Illumina BeadStudio™ software version 3.1.3.0, which calculated CNV data from Intensity and B allele frequency. The calculated results were then exported to Partek Genomics Suite software. Chromosome abnormalities were identified by the cnvPartition algorithm using the default threshold provided by the BeadStudio software and finally visualized by the Partek Genomics Suite v6.4 http://www.partek.com/. A copy number baseline dataset containing 125 Human 1 M HapMap samples (generated by the Partek Inc.) was used to identify aberrant chromosomal regions in GCTs.

### Gene expression microarray probe preparation and data analysis

Total RNA collection, cRNA probe preparation, array hybridization and data analysis were done as described previously [59]. In brief, fresh tissues were immersed in Trizol™ solution (Invitrogen Inc., Carlsbad, CA, USA) and total RNA, including the small RNA fraction, were extracted and precipitated according to the manufacture's instructions. RMA log expression units were calculated from Affymetrix™ HG-U133 Plus 2.0 whole genome array data using the 'affy' package included in the Bioconductor http://www.bioconductor.org suite of software for the R statistical programming language http://www.r-project.org. The default RMA settings were used to background correct, normalize and summarize all expression values. Significant differences between the sample groups was identified using the '*limma*' (Linear Models for Microarray Analysis) package of the Bioconductor suite, and an empirical Bayesian moderated t-statistic hypothesis test between the two specified phenotypic groups was performed [60]. To control for multiple testing errors, we then applied a false discovery rate algorithm to these *p *values in order to calculate a set of *q *values, thresholds of the expected proportion of false positives, or false rejections of the null hypothesis [61].

Heat maps were created by the dChip software http://www.dchip.org/. Classical multidimensional scaling (MDS) was performed using the standard function of the R program to provide a visual impression of how the various sample groups are related. Gene annotation was performed by the ArrayFusion web tool http://microarray.ym.edu.tw/tools/arrayfusion/[62]. Gene enrichment analysis was performed by the Gene Ontology (GO) and KEGG databases using the WebGestalt http://bioinfo.vanderbilt.edu/webgestalt/[41] and DAVID Bioinformatics Resources 2008 http://david.abcc.ncifcrf.gov/[63] interfaces, respectively. The Euclidean distance between two groups of samples is calculated by the average linkage measure (the mean of all pair-wise distances (linkages) between members of the two groups concerned) [59]. The standard error of the average linkage distance between two groups (the standard deviation of pair-wise linkages divided by the square root of the number of linkages) is quoted when inter-group distances are compared in the text.

### Real-time quantitative polymerase chain reaction

Between 100 ng to 1 μg of total RNA was used to perform reverse transcription (RT) using the RevertAid™ Reverse transcriptase kit (Cat. K1622; Fermentas, Glen Burnie, Maryland, USA) as directed by the manufacturer. Real-time PCR reactions were performed using Maxima™ SYBR Green qPCR Master Mix (Cat. K0222; Fermentas, Glen Burnie, Maryland, USA), and the specific products were detected and analyzed using the StepOne™ sequence detector (Applied Biosystems, USA). The expression level of each microRNA was normalized to the expression level of U6 small nuclear RNA, while the expression level of each gene was normalized to GAPDH expression. For hsa-miR-142-5p, the forward primer was 5'-CGCCGGCATAAAGTAGAAAGC-3' and the reverse transcription primer was 5'-GTCGTATCCAGTGCAGGGTCCGAGGTATTCGCACTGGATACGACAGTAGT-3'. For hsa-miR-335, the forward primer was 5'-GGCGTCAAGAGCAATAACGAA-3' and the reverse transcription primer was 5'-GTCGTATCCAGTGCAGGGTCCGAGGTATTCGCACTGGATACGACACATTT-3'. For has-miR-654-3p, the forward primer was 5'-GCGCTATGTCTGCTGACCAT-3' and the reverse transcription primer was 5'-GTCGTATCCAGTGCAGGGTCCGAGGTATTCGCACTGGATACGAAAGGTG-3'. For U6, the forward primer was 5'-CTCGCTTCGGCAGCAC-3' and the reverse primer was 5'-AACGCTTCACGAATTTGCG-'3'. For NANOG, the forward primer was 5'-AGAACTCTCCAACATCCTGAACCT-3' and the reverse primer was 5'-TGCCACCTCTTAGATTTCATTCTCT-3'. For SNAI2 (alias SLUG), the forward primer was 5'-TGACAGGCATGGAGTAACTCTCA-3' and the reverse primer was 5'-AAATGCTGGAGAACTGGAAAG-3'. For POU5F1 (alias OCT4), the forward primer was 5'-CGGAGGAGTCCCAGGACAT-3' and the reverse primer was 5'-CCCACATCGGCCTGTGTATAT. For GAPDH, the forward primer was 5'-CCAGCCGAGCCACATCGCTC-3' and the reverse primer was 5'-ATGAGCCCCAGCCTTCTCCAT-3'.

## Competing interests

The authors declare that they have no competing interests.

## Authors' contributions

HWW, YHW, and TTW conceived the study and identified its value to pediatric tumor research. HWW and YHW designed the analysis approach. MLL, MEC, DJL, and TTW collected the tumor samples. HWW, YHW, and JYH carried out the implementation of data analysis. HWW, MTH, and TTW provided biological guidance during the analysis process. The manuscript was written by HWW and YHW, and all authors read and approved the final manuscript.

## Supplementary Material

Additional file 1 **Summary of patient details and microarray data**. **(A) **Classification, age distribution, gender ratio, and percentage of specific types of primary pediatric intracranial germ cell tumors from Taiwan. **(B) **Clinical data for the 21 cases of primary pediatric CNS GCTs used for genomics studies at Taipei VGH. **(C) **Tumor characteristics between the extra CNS GCT study (Korkola *et al*.) and CNS GCT study in this report. Click here for file

Additional file 2 **Enumeration of differentially expressed probe sets in germinoma and NGMGCT, respectively**. 13 cases were used to analyze the probe sets specifically enriched in the germinoma group or NGMGCT group (*q *< 0.001). Click here for file

Additional file 3 **Distribution of TW NGMGCT genes in the Wnt signaling pathway**. Schematic representation of Wnt signaling pathway is obtained from KEGG pathway database http://www.genome.jp/kegg/. The locations of the signature genes are labeled by asterisks. Click here for file

Additional file 4 **Distribution of TW NGMGCT genes in the focal adhesion pathway**. Schematic representation of focal adhesion pathway is obtained from KEGG pathway database http://www.genome.jp/kegg/. The locations of the signature genes are labeled by asterisks. Click here for file

Additional file 5 **Summary of the aberrant chromosome regions in each tested individuals**. 16 cases were used to compare the chromosome abnormality between different prognosis groups. Detail data including start point, end point, cytoband, size, and *p *value were listed. Click here for file

## References

[B1] Kuratsu J Ushio Y Epidemiological study of primary intracranial tumors in childhood. A population-based survey in Kumamoto Prefecture, Japan Pediatr Neurosurg 1996 25 5 240 246 discussion 247 10.1159/000121132 9309787

[B2] Rickert CH Paulus W Epidemiology of central nervous system tumors in childhood and adolescence based on the new WHO classification Childs Nerv Syst 2001 17 9 503 511 10.1007/s003810100496 11585322

[B3] Cho KT Wang KC Kim SK Shin SH Chi JG Cho BK Pediatric brain tumors: statistics of SNUH, Korea (1959-2000) Childs Nerv Syst 2002 18 1-2 30 37 10.1007/s00381-001-0547-y 11935241

[B4] Committee of Brain Tumor Registry of J Report of Brain Tumor Registry of Japan (1969-1996) Neurol Med Chir (Tokyo) 2003 43 Suppl i vii 1-111 14705327

[B5] Wong TT Ho DM Chang KP Yen SH Guo WY Chang FC Liang ML Pan HC Chung WY Primary pediatric brain tumors: statistics of Taipei VGH, Taiwan (1975-2004) Cancer 2005 104 10 2156 2167 10.1002/cncr.21430 16220552

[B6] Matsutani M Japanese Pediatric Brain Tumor Study Group Combined chemotherapy and radiation therapy for CNS germ cell tumors--the Japanese experience J Neurooncol 2001 54 3 311 316 10.1023/A:1012743707883 11767296

[B7] Echevarria ME Fangusaro J Goldman S Pediatric central nervous system germ cell tumors: a review Oncologist 2008 13 6 690 699 10.1634/theoncologist.2008-0037 18586924

[B8] Matsutani M Clinical management of primary central nervous system germ cell tumors Semin Oncol 2004 31 5 676 683 10.1053/j.seminoncol.2004.07.010 15497121

[B9] Matsutani M Sano K Takakura K Fujimaki T Nakamura O Funata N Seto T Primary intracranial germ cell tumors: a clinical analysis of 153 histologically verified cases J Neurosurg 1997 86 3 446 455 10.3171/jns.1997.86.3.0446 9046301

[B10] Sano K Matsutani M Seto T So-called intracranial germ cell tumours: personal experiences and a theory of their pathogenesis Neurol Res 1989 11 2 118 126 2569683 10.1080/01616412.1989.11739874

[B11] Packer RJ Cohen BH Cooney K Intracranial germ cell tumors Oncologist 2000 5 4 312 320 10964999

[B12] Sperger JM Chen X Draper JS Antosiewicz JE Chon CH Jones SB Brooks JD Andrews PW Brown PO Thomson JA Gene expression patterns in human embryonic stem cells and human pluripotent germ cell tumors Proc Natl Acad Sci USA 2003 100 23 13350 13355 10.1073/pnas.2235735100 14595015 PMC263817

[B13] Werbowetski-Ogilvie TE Bhatia M Pluripotent human stem cell lines: what we can learn about cancer initiation Trends Mol Med 2008 14 8 323 332 10.1016/j.molmed.2008.06.005 18635398

[B14] Josephson R Ording CJ Liu Y Shin S Lakshmipathy U Toumadje A Love B Chesnut JD Andrews PW Rao MS Auerbach JM Qualification of embryonal carcinoma 2102Ep as a reference for human embryonic stem cell research Stem Cells 2007 25 2 437 446 10.1634/stemcells.2006-0236 17284651

[B15] Walsh J Andrews PW Expression of Wnt and Notch pathway genes in a pluripotent human embryonal carcinoma cell line and embryonic stem cell APMIS 2003 111 1 197 210 discussion 210-191 10.1034/j.1600-0463.2003.1110124.x 12760378

[B16] Korkola JE Houldsworth J Chadalavada RS Olshen AB Dobrzynski D Reuter VE Bosl GJ Chaganti RS Down-regulation of stem cell genes, including those in a 200-kb gene cluster at 12p13.31, is associated with in vivo differentiation of human male germ cell tumors Cancer Res 2006 66 2 820 827 10.1158/0008-5472.CAN-05-2445 16424014

[B17] Palmer RD Barbosa-Morais NL Gooding EL Muralidhar B Thornton CM Pett MR Roberts I Schneider DT Thorne N Tavare S Nicholson JC Coleman N Children's Cancer and Leukaemia Group Pediatric malignant germ cell tumors show characteristic transcriptome profiles Cancer Res 2008 68 11 4239 4247 10.1158/0008-5472.CAN-07-5560 18519683

[B18] Lim LP Lau NC Garrett-Engele P Grimson A Schelter JM Castle J Bartel DP Linsley PS Johnson JM Microarray analysis shows that some microRNAs downregulate large numbers of target mRNAs Nature 2005 433 7027 769 773 10.1038/nature03315 15685193

[B19] Baek D Villen J Shin C Camargo FD Gygi SP Bartel DP The impact of microRNAs on protein output Nature 2008 455 7209 64 71 10.1038/nature07242 18668037 PMC2745094

[B20] Selbach M Schwanhausser B Thierfelder N Fang Z Khanin R Rajewsky N Widespread changes in protein synthesis induced by microRNAs Nature 2008 455 7209 58 63 10.1038/nature07228 18668040

[B21] Ferretti E De Smaele E Po A Di Marcotullio L Tosi E Espinola MS Di Rocco C Riccardi R Giangaspero F Farcomeni A Nofroni I Laneve P Gioia U Caffarelli E Bozzoni I Screpanti I Gulino A MicroRNA profiling in human medulloblastoma Int J Cancer 2009 124 3 568 577 10.1002/ijc.23948 18973228

[B22] Ferretti E De Smaele E Miele E Laneve P Po A Pelloni M Paganelli A Di Marcotullio L Caffarelli E Screpanti I Bozzoni I Gulino A Concerted microRNA control of Hedgehog signalling in cerebellar neuronal progenitor and tumour cells EMBO J 2008 27 19 2616 2627 10.1038/emboj.2008.172 18756266 PMC2567402

[B23] Chen Y Stallings RL Differential patterns of microRNA expression in neuroblastoma are correlated with prognosis, differentiation, and apoptosis Cancer Res 2007 67 3 976 983 10.1158/0008-5472.CAN-06-3667 17283129

[B24] Gillis AJ Stoop HJ Hersmus R Oosterhuis JW Sun Y Chen C Guenther S Sherlock J Veltman I Baeten J Spek PJ van der de Alarcon P Looijenga LH High-throughput microRNAome analysis in human germ cell tumours J Pathol 2007 213 3 319 328 10.1002/path.2230 17893849

[B25] Summersgill B Goker H Weber-Hall S Huddart R Horwich A Shipley J Molecular cytogenetic analysis of adult testicular germ cell tumours and identification of regions of consensus copy number change Br J Cancer 1998 77 2 305 313 9461002 10.1038/bjc.1998.47 PMC2151231

[B26] Moran CA Suster S Koss MN Primary germ cell tumors of the mediastinum: III. Yolk sac tumor, embryonal carcinoma, choriocarcinoma, and combined nonteratomatous germ cell tumors of the mediastinum--a clinicopathologic and immunohistochemical study of 64 cases Cancer 1997 80 4 699 707 10.1002/(SICI)1097-0142(19970815)80:4<699::AID-CNCR8>3.0.CO;2-I 9264353

[B27] Mostert M Rosenberg C Stoop H Schuyer M Timmer A Oosterhuis W Looijenga L Comparative genomic and in situ hybridization of germ cell tumors of the infantile testis Lab Invest 2000 80 7 1055 1064 10908150 10.1038/labinvest.3780110

[B28] van Echten J Timmer A Veen AY van der Molenaar WM de Jong B Infantile and adult testicular germ cell tumors. a different pathogenesis? Cancer Genet Cytogenet 2002 135 1 57 62 10.1016/S0165-4608(01)00643-4 12072204

[B29] Zahn S Sievers S Alemazkour K Orb S Harms D Schulz WA Calaminus G Gobel U Schneider DT Imbalances of chromosome arm 1p in pediatric and adult germ cell tumors are caused by true allelic loss: a combined comparative genomic hybridization and microsatellite analysis Genes Chromosomes Cancer 2006 45 11 995 1006 10.1002/gcc.20363 16897744

[B30] Palmer RD Foster NA Vowler SL Roberts I Thornton CM Hale JP Schneider DT Nicholson JC Coleman N Malignant germ cell tumours of childhood: new associations of genomic imbalance Br J Cancer 2007 96 4 667 676 10.1038/sj.bjc.6603602 17285132 PMC2360055

[B31] Kader AK Liu J Shao L Dinney CP Lin J Wang Y Gu J Grossman HB Wu X Matrix metalloproteinase polymorphisms are associated with bladder cancer invasiveness Clin Cancer Res 2007 13 9 2614 2620 10.1158/1078-0432.CCR-06-1187 17473191

[B32] Kawase A Ishii G Nagai K Ito T Nagano T Murata Y Hishida T Nishimura M Yoshida J Suzuki K Ochiai A Podoplanin expression by cancer associated fibroblasts predicts poor prognosis of lung adenocarcinoma Int J Cancer 2008 123 5 1053 1059 10.1002/ijc.23611 18546264

[B33] Takahashi K Yamanaka S Induction of pluripotent stem cells from mouse embryonic and adult fibroblast cultures by defined factors Cell 2006 126 4 663 676 10.1016/j.cell.2006.07.024 16904174

[B34] Yu J Vodyanik MA Smuga-Otto K Antosiewicz-Bourget J Frane JL Tian S Nie J Jonsdottir GA Ruotti V Stewart R Slukvin II Thomson JA Induced pluripotent stem cell lines derived from human somatic cells Science 2007 318 5858 1917 1920 10.1126/science.1151526 18029452

[B35] Katoh Y Katoh M Hedgehog signaling, epithelial-to-mesenchymal transition and miRNA (review) Int J Mol Med 2008 22 3 271 275 18698484

[B36] Ben-Porath I Thomson MW Carey VJ Ge R Bell GW Regev A Weinberg RA An embryonic stem cell-like gene expression signature in poorly differentiated aggressive human tumors Nat Genet 2008 40 5 499 507 10.1038/ng.127 18443585 PMC2912221

[B37] Parathath SR Mainwaring LA Fernandez LA Campbell DO Kenney AM Insulin receptor substrate 1 is an effector of sonic hedgehog mitogenic signaling in cerebellar neural precursors Development 2008 135 19 3291 3300 10.1242/dev.022871 18755774 PMC2673703

[B38] Rubin R Arzumanyan A Soliera AR Ross B Peruzzi F Prisco M Insulin receptor substrate (IRS)-1 regulates murine embryonic stem (mES) cells self-renewal J Cell Physiol 2007 213 2 445 453 10.1002/jcp.21185 17620314 PMC3760688

[B39] Pinson L Auge J Audollent S Mattei G Etchevers H Gigarel N Razavi F Lacombe D Odent S Le Merrer M Amiel J Munnich A Meroni G Lyonnet S Vekemans M Attie-Bitach T Embryonic expression of the human MID1 gene and its mutations in Opitz syndrome J Med Genet 2004 41 5 381 386 10.1136/jmg.2003.014829 15121778 PMC1735763

[B40] Harris MA Clark J Ireland A Lomax J Ashburner M Foulger R Eilbeck K Lewis S Marshall B Mungall C Richter J Rubin GM Blake JA Bult C Dolan M Drabkin H Eppig JT Hill DP Ni L Ringwald M Balakrishnan R Cherry JM Christie KR Costanzo MC Dwight SS Engel S Fisk DG Hirschman JE Hong EL Nash RS Sethuraman A Theesfeld CL Botstein D Dolinski K Feierbach B Berardini T Mundodi S Rhee SY Apweiler R Barrell D Camon E Dimmer E Lee V Chisholm R Gaudet P Kibbe W Kishore R Schwarz EM Sternberg P Gwinn M Hannick L Wortman J Berriman M Wood V de la Cruz N Tonellato P Jaiswal P Seigfried T White R Gene Ontology Consortium The Gene Ontology (GO) database and informatics resource Nucleic Acids Res 2004 32 Database D258 261 14681407 10.1093/nar/gkh036 PMC308770

[B41] Zhang B Kirov S Snoddy J WebGestalt: an integrated system for exploring gene sets in various biological contexts Nucleic Acids Res 2005 33 Web Server W741 748 10.1093/nar/gki475 15980575 PMC1160236

[B42] Moran CA Suster S Przygodzki RM Koss MN Primary germ cell tumors of the mediastinum: II. Mediastinal seminomas--a clinicopathologic and immunohistochemical study of 120 cases Cancer 1997 80 4 691 698 10.1002/(SICI)1097-0142(19970815)80:4<691::AID-CNCR7>3.0.CO;2-Q 9264352

[B43] Ulbright TM Germ cell tumors of the gonads: a selective review emphasizing problems in differential diagnosis, newly appreciated, and controversial issues Mod Pathol 2005 18 Suppl 2 S61 79 10.1038/modpathol.3800310 15761467

[B44] Chang TC Wentzel EA Kent OA Ramachandran K Mullendore M Lee KH Feldmann G Yamakuchi M Ferlito M Lowenstein CJ Arking DE Beer MA Maitra A Mendell JT Transactivation of miR-34a by p53 broadly influences gene expression and promotes apoptosis Mol Cell 2007 26 5 745 752 10.1016/j.molcel.2007.05.010 17540599 PMC1939978

[B45] Liu T Papagiannakopoulos T Puskar K Qi S Santiago F Clay W Lao K Lee Y Nelson SF Kornblum HI Doyle F Petzold L Shraiman B Kosik KS Detection of a microRNA signal in an in vivo expression set of mRNAs PLoS ONE 2007 2 8 e804 10.1371/journal.pone.0000804 17726534 PMC1950084

[B46] Porkka KP Pfeiffer MJ Waltering KK Vessella RL Tammela TL Visakorpi T MicroRNA expression profiling in prostate cancer Cancer Res 2007 67 13 6130 6135 10.1158/0008-5472.CAN-07-0533 17616669

[B47] Almstrup K Hoei-Hansen CE Wirkner U Blake J Schwager C Ansorge W Nielsen JE Skakkebaek NE Rajpert-De E Meyts Leffers H Embryonic stem cell-like features of testicular carcinoma in situ revealed by genome-wide gene expression profiling Cancer Res 2004 64 14 4736 4743 10.1158/0008-5472.CAN-04-0679 15256440

[B48] de Jong J Looijenga LH Stem cell marker OCT3/4 in tumor biology and germ cell tumor diagnostics: history and future Crit Rev Oncog 2006 12 3-4 171 203 17425502 10.1615/critrevoncog.v12.i3-4.10

[B49] Shi Y Desponts C Do JT Hahm HS Scholer HR Ding S Induction of pluripotent stem cells from mouse embryonic fibroblasts by Oct4 and Klf4 with small-molecule compounds Cell Stem Cell 2008 3 5 568 574 10.1016/j.stem.2008.10.004 18983970

[B50] Mani SA Guo W Liao MJ Eaton EN Ayyanan A Zhou AY Brooks M Reinhard F Zhang CC Shipitsin M Campbell LL Polyak K Brisken C Yang J Weinberg RA The epithelial-mesenchymal transition generates cells with properties of stem cells Cell 2008 133 4 704 715 10.1016/j.cell.2008.03.027 18485877 PMC2728032

[B51] Fritsch MK Schneider DT Schuster AE Murdoch FE Perlman EJ Activation of Wnt/beta-catenin signaling in distinct histologic subtypes of human germ cell tumors Pediatr Dev Pathol 2006 9 2 115 131 10.2350/08-05-0097.1 16822086

[B52] Korkola JE Houldsworth J Feldman DR Olshen AB Qin LX Patil S Reuter VE Bosl GJ Chaganti RS Identification and validation of a gene expression signature that predicts outcome in adult men with germ cell tumors J Clin Oncol 2009 27 31 5240 5247 10.1200/JCO.2008.20.0386 19770384 PMC3651602

[B53] Kanetsky PA Mitra N Vardhanabhuti S Li M Vaughn DJ Letrero R Ciosek SL Doody DR Smith LM Weaver J Albano A Chen C Starr JR Rader DJ Godwin AK Reilly MP Hakonarson H Schwartz SM Nathanson KL Common variation in KITLG and at 5q31.3 predisposes to testicular germ cell cancer Nat Genet 2009 41 7 811 815 10.1038/ng.393 19483682 PMC2865677

[B54] Rapley EA Turnbull C Al Olama AA Dermitzakis ET Linger R Huddart RA Renwick A Hughes D Hines S Seal S Morrison J Nsengimana J Deloukas P UK Testicular Cancer Collaboration Rahman N Bishop DT Easton DF Stratton MR A genome-wide association study of testicular germ cell tumor Nat Genet 2009 41 7 807 810 10.1038/ng.394 19483681 PMC2871592

[B55] Stock C Ambros IM Lion T Haas OA Zoubek A Gadner H Ambros PF Detection of numerical and structural chromosome abnormalities in pediatric germ cell tumors by means of interphase cytogenetics Genes Chromosomes Cancer 1994 11 1 40 50 10.1002/gcc.2870110107 7529045

[B56] Kraggerud SM Szymanska J Abeler VM Kaern J Eknaes M Heim S Teixeira MR Trope CG Peltomaki P Lothe RA DNA copy number changes in malignant ovarian germ cell tumors Cancer Res 2000 60 11 3025 3030 10850452

[B57] Huang TS Hsieh JY Wu YH Jen CH Tsuang YH Chiou SH Partanen J Anderson H Jaatinen T Yu YH Wang HW Functional network reconstruction reveals somatic stemness genetic maps and dedifferentiation-like transcriptome reprogramming induced by GATA2 Stem Cells 2008 26 5 1186 1201 10.1634/stemcells.2007-0821 18308945

[B58] Li SS Liu YH Tseng CN Chung TL Lee TY Singh S Characterization and gene expression profiling of five new human embryonic stem cell lines derived in Taiwan Stem Cells Dev 2006 15 4 532 555 10.1089/scd.2006.15.532 16978057

[B59] Wang HW Trotter MW Lagos D Bourboulia D Henderson S Makinen T Elliman S Flanagan AM Alitalo K Boshoff C Kaposi sarcoma herpesvirus-induced cellular reprogramming contributes to the lymphatic endothelial gene expression in Kaposi sarcoma Nat Genet 2004 36 7 687 693 10.1038/ng1384 15220918

[B60] Smyth GK Linear models and empirical bayes methods for assessing differential expression in microarray experiments Stat Appl Genet Mol Biol 2004 3 1 Article3 16646809 10.2202/1544-6115.1027

[B61] Storey JD Tibshirani R Statistical significance for genomewide studies Proc Natl Acad Sci USA 2003 100 16 9440 9445 Epub 2003 Jul 9425 10.1073/pnas.1530509100 12883005 PMC170937

[B62] Yang TP Chang TY Lin CH Hsu MT Wang HW ArrayFusion: a web application for multi-dimensional analysis of CGH, SNP and microarray data Bioinformatics 2006 22 21 2697 2698 10.1093/bioinformatics/btl457 16935928

[B63] Dennis G Jr Sherman BT Hosack DA Yang J Gao W Lane HC Lempicki RA DAVID: Database for Annotation, Visualization, and Integrated Discovery Genome Biol 2003 4 5 P3 10.1186/gb-2003-4-5-p3 12734009 PMC193660

