# Supplementary material for: Pediatric primary central nervous system germ cell tumors of different prognosis groups show characteristic miRNome traits and chromosome copy number variations
Source: BMC Genomics. 2010 Feb 24;11:132. doi: 10.1186/1471-2164-11-132 (PMC2837036; doi:10.1186/1471-2164-11-132)
Supplement: Additional file 1 — Summary of patient details and microarray data. (A) Classification, age distribution, gender ratio, and percentage of specific types of primary pediatric intracranial germ cell tumors from Taiwan. (B) Clinical data for the 21 cases of primary pediatric CNS GCTs used for genomics studies at Taipei VGH. (C) Tumor characteristics between the extra CNS GCT study (Korkola et al.) and CNS GCT study in this report. [file 1471-2164-11-132-S1.PDF]

(A)

Classification, age distribution, gender ratio, and percentage of specific types of primary pediatric intracranial germ cell tumors in child (N = 176/1186 cases)

Type of tumor	Mean age (range)	Sex ratio (M/F)	No. of cases	Percentage with histological diagnosis	Percentage N=176
Germinoma	11.9 yrs (1.3-18 yrs)	4.2	103	62.1	58.5
Mature teratoma	7.6yrs	9/0	9	100	5.1
NGMGCT	9.6 yrs (7 days–16.5 yrs)	2.4	62	90.3	35.2
Immature teratoma	6.9 yrs (7 days-14.5 yrs)	5.3	21	100	11.9
Mixed GCT	10.3 yrs (1.3-16.4 yrs)	2.3	23	100	13.1%
Yolk sac tumor, pure	10.0 yrs (2.6-14.5 yrs)	1	10	100	5.7%
Diagnosed by markers	14.3 yrs (7.4-16.5 yrs)	2	6	0	3.4%
Unclassified GCT	8.5 yrs (5.8-10.9 yrs)	2/0	3	100	1.7%

(B)

Clinical data for the 21 cases of primary pediatric CNS GCTs used for genomics studies at Taipei VGH

Case	Types of GCT	Location	Age	Sex (M/F)	Treatment	Outcome FU period	mRNA	miRNA	CNV
1	Germinoma	Basal ganglia	15.9 yrs	M	STR + RT	Alive 2.1 yrs	V	V	V
2	Germinoma	Suprasellar	10.4 yrs	F	STR + RT	Alive 1.9 yrs	V	V	V
3	Germinoma	Suprasellar	14.2 yrs	M	STR +RT	Alive 12 mos.	V	V	V
4	Germinoma	Supasellar	10.4 yrs	M	PR + RT	Alive 8 mos.	V	V	(-)
5	Germinoma	Supasellar	11.5yrs	M	STR+RT	Alive 6 mos	V	V	(-)
6	Germinoma	Suprasellar+3rd V	13.6yrs	M	PR+RT	Alive 2.8yrs	(-)	(-)	V
7	Germinoma	Suprasellar	12yrs	F	PR+RT	Alive 2.6yrs	(-)	(-)	V
8	Germinoma	Basal ganglia	12.2yrs	M	NTR+RT	Alive 4.5yrs	(-)	(-)	V
9	Germinoma	Left hypothalamic	14.2yrs	M	NTR+RT	Alive 4.1yrs	(-)	(-)	V

10	MT	Pineal	4.6yrs	M	NTR	Alive 8.75yrs	(-)	(-)	V
11	MT (spinal tumor)	intramedullar	4mos	F	PR	Alive 9mos	(-)	(-)	V
12	Germinoma 20% + MT 80%	Pineal	9.7 yrs	M	NTR + RT	Alive 1.25yrs	V	(-)	(-)
13	MT + ↑ AFP	Pineal	11.5yrs	M	RT+CMT	Alive 1.5yrs	(-)	(-)	V
14	Germinoma + ↑ AFP	Pineal	14 yrs	M	NTR+RT+CMT	Alive 4 mos	V	V	(-)
15	Germinoma + IMT	Pineal	4.3 yrs	M	GTR	Alive 1.7 yrs	V	V	(-)
16	Germinoma + IMT	Pineal	10.25yrs	M	NTR+RT+CMT	Alive 5mos	V	V	(-)
17	Germinoma + IMT	Basal ganglia	11.5yrs	M	STR+RT+CMT	Alive 8.6yrs	(-)	(-)	V
18	IMT	Frontal lobe	10 days	F	GRR	Alive 1.4 yrs	V	V	V
19	Germinoma + YST	Pineal	14.5 yrs	M	PR + CSI +	Died	V	V	V

	(dominant)				Salvage CMT Early recurrence	6 months			
20	IMT + YST (predominant)	Suprasellar	10.6 yrs	M	PR + CSI + CMT	Alive 7.4 yrs	V	V	V
21	IT + EC + YST (dominant)	Pineal + 3rd V	7.7 yrs	M	GTR + RT + CMT	Alive 1.3 yrs	V	V	V

RT: radiotherapy; ELF: extended local field radiation; CMT: chemotherapy; CSI: craniospinal axis irradiation

MT: mature teratoma; IMT: immature teratoma; YST: yolk sac tumor; EC: embryonal carcinoma

(C)

Tumor characteristics between the extra CNS GCT study (Korkola et al.) and CNS GCT study in this report

Characteristic	Kordola et al.		Taipei VGH	
	Training set<br>No. (%)	Validation set<br>No. (%)	Series<br>No. (%)	Study group<br>No. (%)
Age, Years – Median	29.0	28.1	11.7	11.6
Range	15.4 – 65.0	16.6 -45.2	7 days - 18	10 days – 15.9
Tumor site	26 (35%)	18 (53%)	176 (100%)	21 (100%)
Primary tumor	17 (23%)	14 (41%)		
Non-CNS	9 (12%)	4 (12%)		
Testis	48 (65%)	16 (47%)		
Mediastinum				
CNS				
Pure histology	16 (22%)	9 (26%)	103 (58.5%)	9 (42.9%)
Pure seminoma (germinoma)			30 (17.0%)	2 (9.5%)
Pure teratoma			9 (5.1%)	1 (4.8%)
mature teratoma			21 (11.9%)	1 (4.8%)
immature teratoma			10 (5.7%)	
Pure YST				

Mixed histology	5 (7%)	2 (6%)	23 (13.1%)	9 (42.9%)***
Seminoma (Germinoma) as part of it	22 (30%)	9 (26%)	15 (8.5%)	6 (28.6%)
Teratoma as part of it			19 (10.8%)	7 (33.3%)
YST as part of it			11 (6.3%)	4 (19.1%)
Pure or dominant (if mixed) histology	5 (7%)	3 (9%)	36 (20.5%)*	4 (19.0%)
CC	19 (26%)	6 (18%)	13 (7.4%)	3 (14.3%)
EC	25 (34%)	12 (35)	3 (1.7%)	
TER	21 (28%)	11 (32%)		
YST	2 (3%)	2 (6%)		
Trophoblast	2 (3%)	0 (0%)		
N/A				
Secondary malignant transformation	5 (7%)	6 (18%)	4 (2.3%)**	
No. of TER with malignant transformation				

* Pure immature teratoma and teratoma dominant teratomas

** 3 immature teratoma and 1 mature teratoma changed to malignant mixed GCTs on recurrence

**1 mixed GCT was defined by germinoma + ↑AFP, 1 mixed GCT was defined as germinoma + mature teratoma
