# Supplementary figures and images for: Pediatric primary central nervous system germ cell tumors of different prognosis groups show characteristic miRNome traits and chromosome copy number variations
Source: BMC Genomics. 2010 Feb 24;11:132. doi: 10.1186/1471-2164-11-132 (PMC2837036; doi:10.1186/1471-2164-11-132)

# WNT SIGNALING PATHWAY

## Canonical pathway

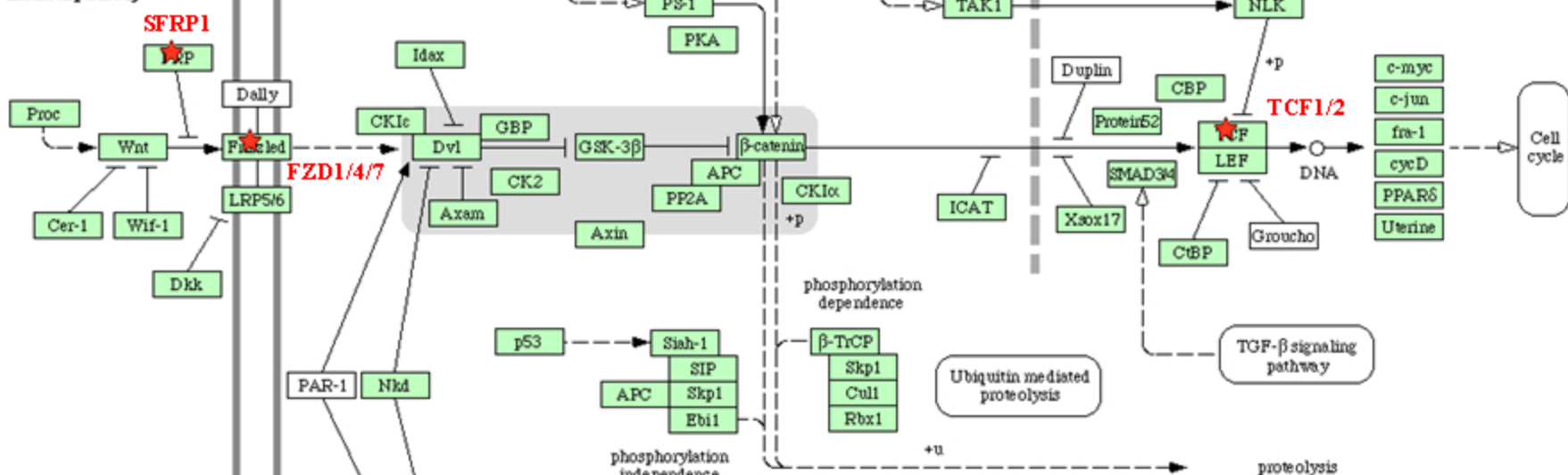

## Planar cell polarity (PCP) pathway

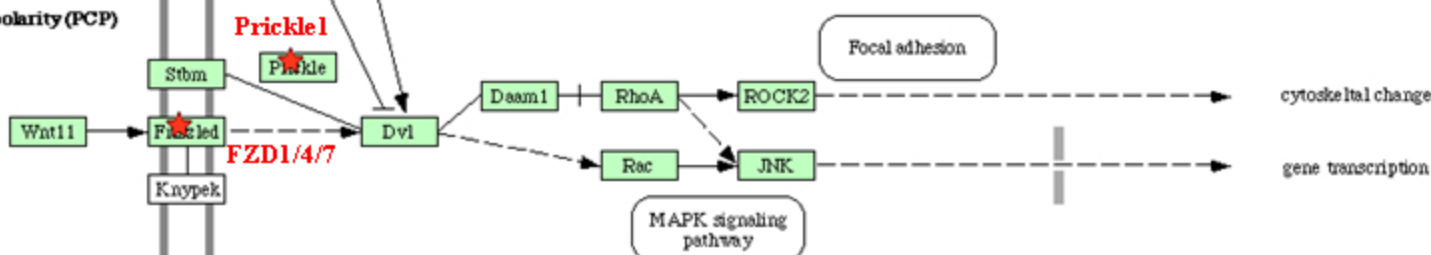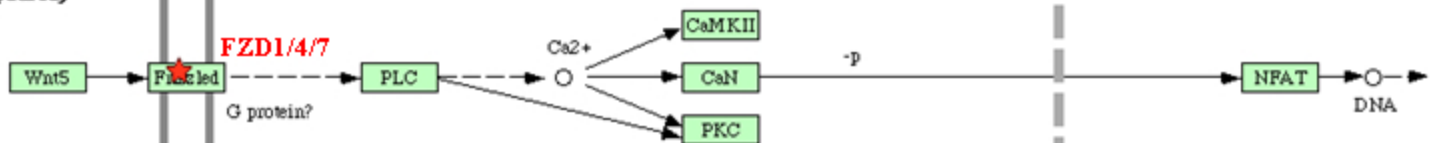

Supplement: Additional file 3 — Distribution of TW NGMGCT genes in the Wnt signaling pathway. Schematic representation of Wnt signaling pathway is obtained from KEGG pathway database http://www.genome.jp/kegg/. The locations of the signature genes are labeled by asterisks. [file 1471-2164-11-132-S3.PDF]

# FOCAL ADHESION

ECM  
(COL1A1 & A2  
COL3A1  
Laminin beta 1  
Thrombospondin 2)

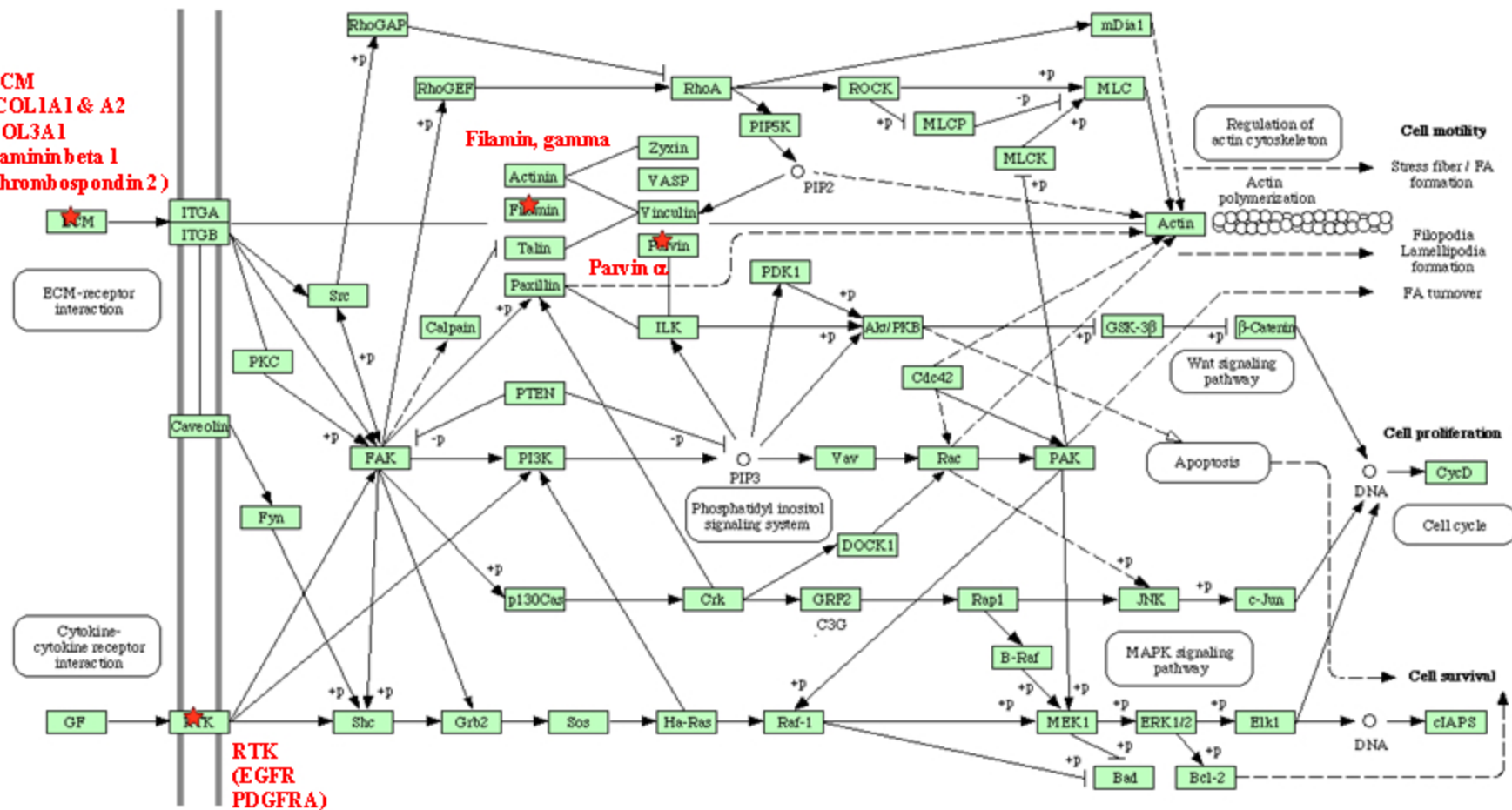

Supplement: Additional file 4 — Distribution of TW NGMGCT genes in the focal adhesion pathway. Schematic representation of focal adhesion pathway is obtained from KEGG pathway database http://www.genome.jp/kegg/. The locations of the signature genes are labeled by asterisks. [file 1471-2164-11-132-S4.PDF]
